# Pro-inflammatory macrophages produce mitochondria-derived superoxide by reverse electron transport at complex I that regulates IL-1β release during NLRP3 inflammasome activation

**DOI:** 10.1038/s42255-025-01224-x

**Published:** 2025-02-19

**Authors:** Alva M. Casey, Dylan G. Ryan, Hiran A. Prag, Suvagata Roy Chowdhury, Eloïse Marques, Keira Turner, Anja V. Gruszczyk, Ming Yang, Dane M. Wolf, Jan Lj. Miljkovic, Joyce Valadares, Patrick F. Chinnery, Richard C. Hartley, Christian Frezza, Julien Prudent, Michael P. Murphy

**Affiliations:** 1 MRC Mitochondrial Biology Unit, Biomedical Campus, University of Cambridge, Cambridge, UK; 2 Department of Medicine, University of Cambridge, Cambridge, UK; 3 Department of Surgery and Cambridge NIHR Biomedical Research Centre, Biomedical Campus, University of Cambridge, Cambridge, UK; 4 University of Cologne, Faculty of Medicine and University Hospital Cologne, Institute for Metabolomics in Ageing, Cluster of Excellence Cellular Stress Responses in Aging-associated Diseases (CECAD), Cologne, Germany; 5 University of Cologne, Faculty of Mathematics and Natural Sciences, Institute of Genetics, Cluster of Excellence Cellular Stress Responses in Aging-associated Diseases (CECAD), Cologne, Germany; 6 WestCHEM School of Chemistry, University of Glasgow, Glasgow, UK

**Keywords:** Metabolomics, Cell signalling, Metabolism, Monocytes and macrophages

## Abstract

Macrophages stimulated by lipopolysaccharide (LPS) generate mitochondria-derived reactive oxygen species (mtROS) that act as antimicrobial agents and redox signals; however, the mechanism of LPS-induced mitochondrial superoxide generation is unknown. Here we show that LPS-stimulated bone-marrow-derived macrophages produce superoxide by reverse electron transport (RET) at complex I of the electron transport chain. Using chemical biology and genetic approaches, we demonstrate that superoxide production is driven by LPS-induced metabolic reprogramming, which increases the proton motive force (∆p), primarily as elevated mitochondrial membrane potential (Δψ_m_) and maintains a reduced CoQ pool. The key metabolic changes are repurposing of ATP production from oxidative phosphorylation to glycolysis, which reduces reliance on F_1_F_O_-ATP synthase activity resulting in a higher ∆p, while oxidation of succinate sustains a reduced CoQ pool. Furthermore, the production of mtROS by RET regulates IL-1β release during NLRP3 inflammasome activation. Thus, we demonstrate that ROS generated by RET is an important mitochondria-derived signal that regulates macrophage cytokine production.

## Abstract (web summary)

Casey et al. explore the basic mechanisms of mitochondrial superoxide production and its impact on interleukin-1β production in LPS-treated macrophages.

## Main

Macrophages are a key component of the innate immune system, acting to both fight infection and to resolve the inflammatory response and promote tissue repair^1^. One weapon in the arsenal of pro-inflammatory macrophages to fight bacterial infection is the production of reactive oxygen species (ROS) and recent studies have shown that mitochondria are key generators of superoxide and hydrogen peroxide in pro-inflammatory macrophages^2^. Furthermore, mitochondria-derived ROS (mtROS) are important as bactericidal agents and have been implicated in the regulation of macrophage cytokine production^2–4^.

Despite the importance of mtROS in macrophage immune function, the mechanism of their production is not understood. A major mechanism of mitochondrial superoxide production is reverse electron transport (RET) at complex I of the electron transport chain^4^. Complex I normally accepts electrons from NADH and pumps protons across the mitochondrial inner membrane to generate the proton motive force (Δp), which drives ATP synthesis. For forward electron transport (FET) to occur the difference in reduction potential between the NAD^+^/NADH and the CoQ/CoQH_2_ couples (ΔE_h_) must be sufficient for two electrons to pump four protons against Δp such that the thermodynamic requirement for FET is 2ΔEh > 4Δp (ref. ^5^). Electrons can also be transferred in reverse, from the CoQ pool through complex I to its flavin mononucleotide (FMN). The electrons can then be transferred from FMN to NAD^+^ or to oxygen to produce superoxide^6^. For RET to occur, 4Δp > 2ΔE_h_ must be satisfied, as is the case in ischaemia/reperfusion injury^7^.

Following Toll-like receptor-4 (TLR4) signalling by lipopolysaccharide (LPS) in macrophages, there are dramatic changes in metabolism, notably the switch from oxidative phosphorylation to glycolysis for ATP production and the accumulation of succinate from the tricarboxylic acid cycle^4,8,9^. While these findings are consistent with RET driving superoxide formation in LPS-stimulated macrophages, the actual mode and source of mtROS production remains uncertain. The way in which LPS-induced metabolic repurposing is linked to mtROS production is also obscure. Furthermore, how elevated mtROS regulates pro-inflammatory macrophage function in conjunction with metabolic signals such as succinate and itaconate accumulation is not known^4,10^. Therefore, we set out to investigate the mechanism of LPS-induced mitochondrial superoxide generation and to determine how this is driven by the metabolic reprogramming that occurs upon LPS stimulation, as well as the role of mtROS in the regulation of macrophage cytokine production.

## Results

### Time dependence of mitochondrial superoxide production

**Fig. 1:**
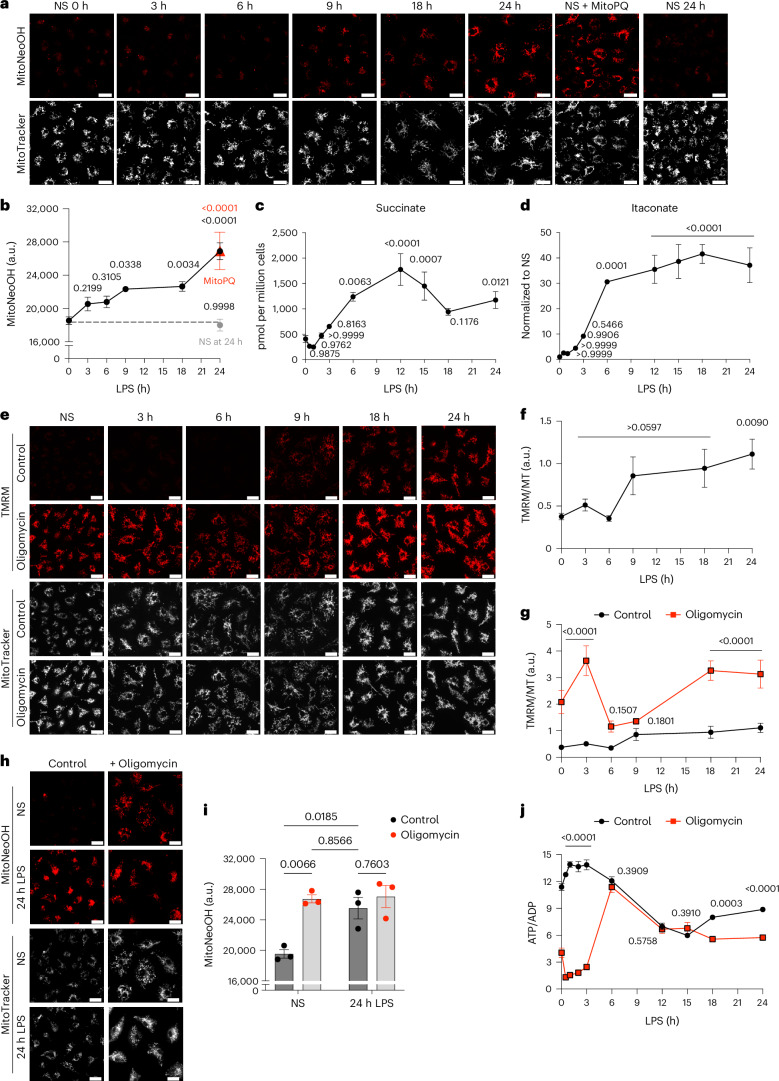
LPS-induced increase in mitochondrial membrane potential drives mitochondrial superoxide production. **a**,**b**, Mitochondrial superoxide measured with MitoNeoD in BMDMs stimulated with LPS for 3–24 h, treated with 10 μM MitoParaquat for 30 min or nonstimulated (NS). NS BMDMs were either left for 24 h before MitoNeoD treatment (NS at 24 h) or immediately treated with MitoNeoD (NS 0 h). Representative images from confocal live-cell imaging show MitoNeoOH, the oxidized product of MitoNeoD and MitoTracker Deep Red FM. Fluorescence intensity of MitoNeoOH, measured as absolute intensity (arbitrary units (a.u.)) (*n* = 3 (NS, 6 h, 9 h, MitoPQ, NS at 24 h), *n* = 6 (3 h, 18 h, 24 h)). **c**,**d**, Succinate and itaconate measured by mass spectrometry in BMDMs treated with LPS for 3–24 h or NS (*n* = 6). **e**–**g**, ∆ψ_m_ measured with TMRM normalized to MitoTracker Deep Red FM in BMDMs stimulated with LPS for 3–24 h or NS and/or treated with 5 μM oligomycin in the last 30 min of LPS treatment or for 30 min in NS cells from confocal live-cell imaging (*n* = 6 (3 h, 6 h, 18 h, 24 h), *n* = 9 (NS, 9 h)) (NS, *P* = 0.00006; 3 h *P* = 9 × 10^−9^; 18 h, *P* = 1 × 10^−5^; 24 h, *P* = 8 × 10^−5^). **h**,**i**, Mitochondrial superoxide production measured with MitoNeoD from confocal live-cell imaging in BMDMs stimulated with LPS for 24 h and/or treated with 5 μM oligomycin in the last 30 min of LPS treatment or for 30 min in NS cells. Fluorescence intensity of MitoNeoOH is measured as absolute intensity (a.u.) (*n* = 6). Scale bars on all representative images, 20 μm. **j**, The ATP/ADP ratio measured per million BMDMs stimulated with LPS for 3–24 h or NS and/or treated with 5 μM oligomycin in the last 30 min of LPS treatment or for 30 min in NS cells (*n* = 3) (NS, 0.5 h, 1 h, 2 h and 3 h, *P* < 1 × 10^−^^15^). All data are mean ± s.e.m. of biological replicates. *P* values displayed above graphs were calculated using two-tailed Student’s *t*-test for paired comparisons or one-way or two-way analysis of variance (ANOVA) for multiple comparisons. Source data

**Extended Data Fig. 1:**
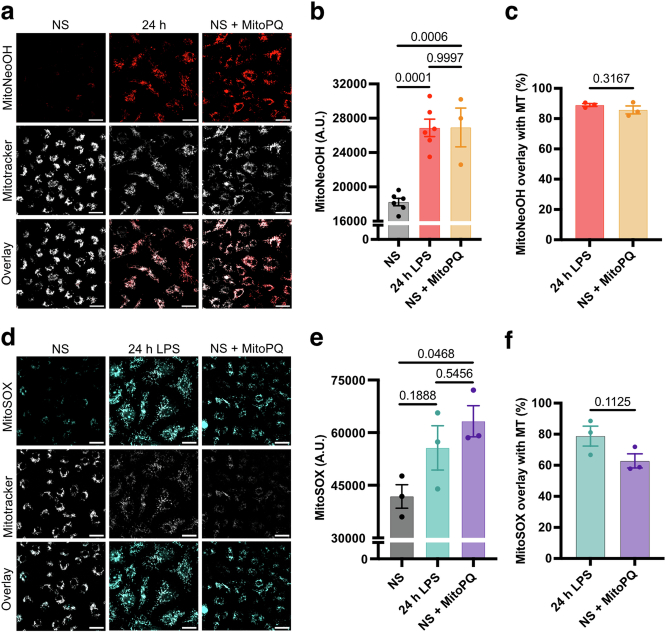
Measurement of mitochondrial superoxide production in BMDMs using MitoNeoD and MitoSOX. **a-c**, Mitochondrial superoxide measured with MitoNeoD in nonstimulated (NS) BMDMs untreated or treated with 10 μM MitoParaquat (MitoPQ) for 30 min or stimulated with LPS for 24 h. **a**, Representative images from confocal live cell imaging show MitoNeoOH, the oxidized product of MitoNeoD and Mitotracker Deep Red FM. **b**, Graph shows fluorescence intensity of MitoNeoOH measured as absolute intensity with Arbitrary Units (A.U.) (n = 3 (NS + MitoPQ), n = 6 (NS, 24 h LPS)). **c**, Quantification of MitoNeoOH fluorescence overlay with MitoTracker Deep Red in BMDMs stimulated with LPS for 24 h and NS BMDMs treated with MitoPQ (n = 3). **d-f**, Mitochondrial superoxide measured with MitoSOX in BMDMs stimulated with LPS for 24 h, NS or treated with 10 μM MitoParaquat for 30 min from confocal live cell imaging. **e**, Fluorescence intensity is measured as absolute intensity (A.U.) (n = 3). **f**, Quantification of MitoSOX fluorescence overlay with MitoTracker Deep Red FM in BMDMs stimulated with LPS for 24 h or NS BMDMs treated with MitoPQ (n = 3). Data are mean +/- S.E.M. of biological replicates. Scale bars on all representative live cell confocal images are 20 μm. *P* values displayed above graphs were calculated using two-tailed Student’s *t*-test for paired comparisons or one-way ANOVA for multiple comparisons. Source data

To determine the mechanism of mitochondrial superoxide production in LPS-stimulated bone-marrow-derived macrophages (BMDMs), we measured mtROS production using the mitochondria-targeted probe MitoNeoD, which is preferentially converted to MitoNeoOH by superoxide and does not intercalate with DNA^11^. This produces a fluorescent signal that is more reflective of superoxide than MitoSOX^11^. MitoNeoOH fluorescence measured in BMDMs stimulated with LPS for 24 h was comparable with acute treatment with the mitochondrial superoxide generator MitoParaquat (MitoPQ), which selectively generates superoxide at complex I^12^, and the LPS-induced MitoNeoOH fluorescence consistently overlayed to greater than 80% with MitoTracker Deep Red (Extended Data Fig. 1a–c). MitoSOX gave a similar outcome as MitoNeoD but the result was more variable and the fluorescence signal overlayed with MitoTracker Deep Red to a lesser degree (Extended Data Fig. 1d–f). Assessment of mitochondrial superoxide production over 24 h following LPS treatment using MitoNeoD showed a gradual increase that reached the same level at 24 h as acute MitoPQ treatment (Fig. 1a,b).

**Extended Data Fig. 2:**
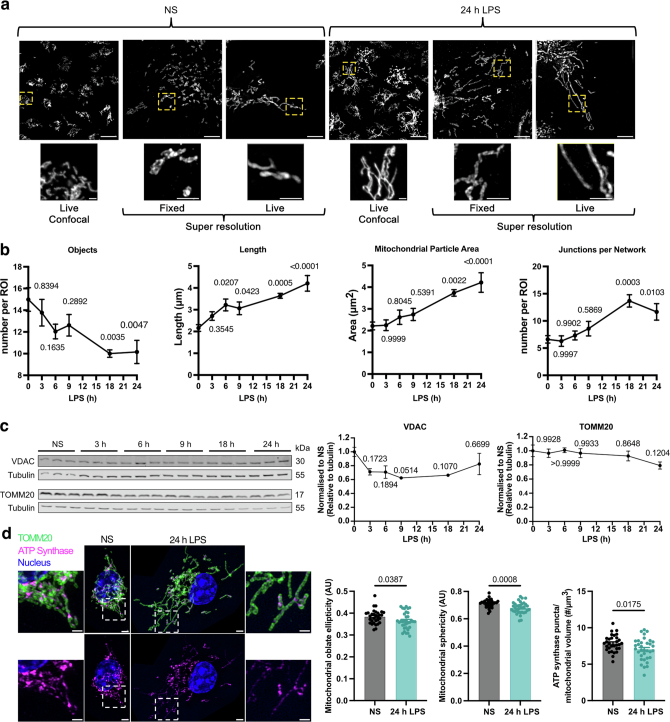
LPS-induced change in mitochondrial morphology. **a**, BMDMs stimulated with LPS for 24 h or nonstimulated (NS). Representative images left to right: Live cell confocal images showing Mitotracker Deep Red FM, fixed cell super-resolution structured illuminated microscopy images showing TOMM20 signal and live cell super-resolution structured illuminated microscopy images showing Mitotracker green. Scale bars are 20 μm for all confocal images and 5 μm for super-resolution images. Scale bars for inset images are 2 μm. **b**, BMDMs were stimulated with LPS for 3–24 h LPS or NS and then treated with 5 nM Mitotracker Deep Red FM for 20 min and imaged by confocal live cell microscopy. Mitochondrial object number, length, area and junctions per network were measured in 20 regions of interest (ROIs) (15 μm^2^) per biological replicates (n = 5 (6 h), n = 6 (3 h, 9 h, 18 h, 24 h), n = 7 (NS)). **c**, Western blot and quantification of VDAC and TOMM20 normalized to tubulin loading control in BMDMs treated with LPS for 3–24 h or NS (n = 3). Blots show three biological replicates for each condition and data are mean normalized to NS and vinculin loading control. **d**, Representative images of BMDMs that were NS or stimulated with LPS for 24 h imaged by structured illumination microscopy (Elyra7) showing ATP synthase (magenta), TOMM20 (green) and nucleus (blue). Scale bars are 2 μm for whole cell images and 1 μm for inset image. Quantifications of mitochondrial morphology and ATP synthase density are mean +/- S.E.M. of whole cells from 3 biological replicates (n = 31 (NS), n = 34 (24 h LPS)). All data are mean +/- S.E.M. of biological replicates. *P* values displayed above graphs calculated using two-tailed Student’s *t*-test for paired comparisons or one-way ANOVA for multiple comparisons. Source data

We next investigated changes in mitochondrial function and morphology to identify the factors that influence mtROS generation. It has been reported that mtROS production following LPS stimulation is associated with increased mitofusin 2 expression^13^ but this contrasts with other studies reporting that LPS induces mitochondrial fragmentation^14,15^. We therefore monitored mitochondrial morphology in BMDMs by both confocal and super-resolution microscopy. These measurements indicated that there were extensive and gradual morphological changes after LPS stimulation, going from punctate to filamentous over 24 h (Extended Data Fig. 2a). This analysis was extended by measuring the number, length and area of mitochondria over time, as well as mitochondrial junctions per network, which indicates the extent of branching, in randomly selected regions of interest (ROIs) (15 μm^2^) (Extended Data Fig. 2b). While the number of mitochondrial particles decreased, mitochondrial length, area and junctions per network increased (Extended Data Fig. 2a,b), corroborating the elongated mitochondrial network observed. These morphological changes did not accompany any change in mitochondrial mass, as determined by VDAC and TOMM20 protein levels (Extended Data Fig. 2c). Mitochondrial architecture was also assessed with structured illumination microscopy in three-dimensions in whole cells to assess mitochondrial ellipticity and sphericity (Extended Data Fig. 2d and Supplementary Videos 1 and 2). Mitochondrial oblate ellipticity and sphericity both decreased with LPS treatment (Extended Data Fig. 2d and Supplementary Videos 1 and 2). This confirmed that nonstimulated macrophages have smaller, shorter and more spherical mitochondria, whereas LPS stimulation results in fewer individual mitochondria, each of which is elongated and more branched. Thus, we show that gradual mitochondrial morphological changes occur in parallel with elevated mitochondrial superoxide production following LPS stimulation.

### Metabolic reprogramming following LPS stimulation

**Extended Data Fig. 3:**
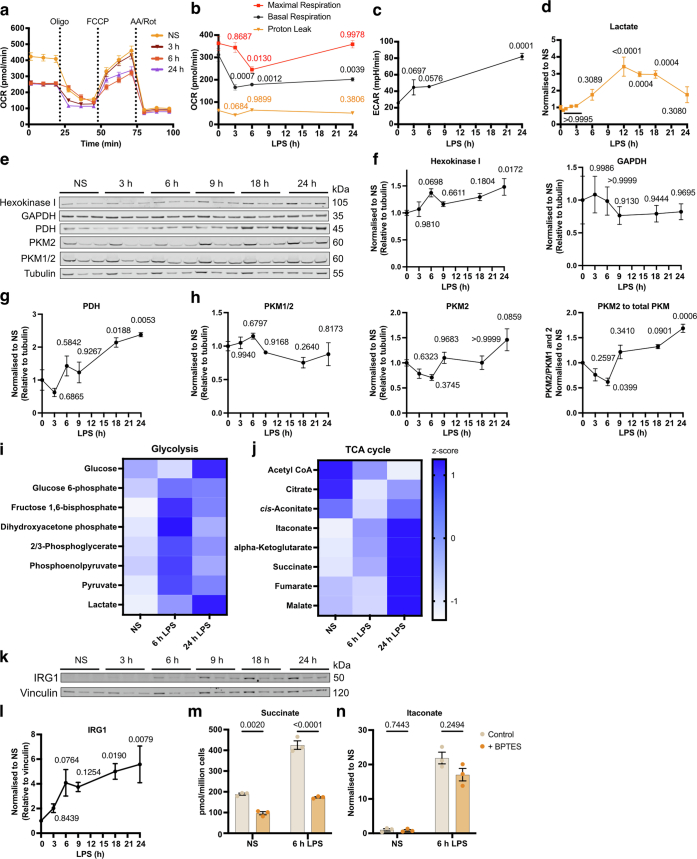
Metabolic reprogramming in LPS-stimulated BMDMs. **a,b**, Oxygen consumption rate (OCR) measured by Seahorse XF mito stress test in nonstimulated (NS) BMDMs and BMDMs stimulated with LPS for 3 h, 6 h or 24 h (n = 3). Data are mean +/- S.E.M. of biological replicates. **c**, Extracellular acidification rate (ECAR) in BMDMs stimulated with LPS for 3 h, 6 h or 24 h or NS measured by Seahorse (n = 3). Data are mean +/- S.E.M. of biological replicates. **d**, Extracellular lactate measured by mass spectrometry in BMDMs stimulated with LPS for 3–24 h or NS (n = 6) (12 h LPS, P = 3×10^-5^). Data are mean +/- S.E.M. of biological replicates. **e**, Western blot of hexokinase I, GAPDH, PDH, PKM2, PKM1/2 and tubulin in BMDMs treated with LPS for 3–24 h or NS (n = 3). Tubulin is a representative blot. Blots show three biological replicates for each condition. **f-h**, Quantification of protein determined by western blotting, normalized to NS and tubulin loading control +/- S.E.M. of biological replicates (n = 3). PKM2 was also normalized to PKM1/2. **i,j**, Metabolites measured by mass spectrometry in BMDMs stimulated with LPS for 6 h or 24 h or nonstimulated (NS) (n = 3). Data depicts z-score of biological replicates. **k,l**, Western blot and quantification of IRG1 normalized to vinculin loading control in BMDMs treated with LPS for 3–24 h or NS (n = 3). Blots show three biological replicates for each condition and data are mean normalized to NS and vinculin loading control. **m,n**, Succinate and itaconate measured by mass spectrometry in BMDMs stimulated with LPS for 6 h or NS, non-treated or treated with 10 μM BPTES for 6 h (n = 3) (6 h LPS Control vs BPTES, P = 1×10^-6^). Data are mean +/- S.E.M. of biological replicates. *P* values displayed above graphs calculated using two-tailed Student’s *t*-test for paired comparisons or one-way ANOVA for multiple comparisons. Source data

To determine the driving forces of mitochondrial superoxide production we investigated changes in macrophage metabolism following LPS stimulation. LPS treatment resulted in decreased mitochondrial respiration (Extended Data Fig. 3a,b), an increase in glycolytic extracellular acidification rate (Extended Data Fig. 3c), and accumulation of lactate, which was secreted from the cell (Extended Data Fig. 3d)^16^. The protein levels of hexokinase I and pyruvate dehydrogenase increased over 24 h LPS treatment, whereas those of GAPDH did not (Extended Data Fig. 3e–g). There was also a switch from pyruvate kinase M1 (PKM1) to PKM2 expression (Extended Data Fig. 3e,h) as has been reported^17^. This was associated with an increase in the level of glycolytic and tricarboxylic acid cycle intermediates (Extended Data Fig. 3i,j). Most notable were the dramatic increases in succinate and itaconate over time (Fig. 1c,d). Itaconate is produced from *cis-*aconitate by immune response gene-1 (IRG1), also known as aconitate decarboxylase 1 (ACOD1)^18^, the expression of which is induced and increases from 6 h LPS treatment (Extended Data Fig. 3k,l). Inhibition of glutaminase with BPTES (bis-2-(5-phenylacetamido-1,3,4-thiadiazol-2-yl)ethyl sulfide) prevented LPS-induced succinate accumulation (Extended Data Fig. 3m)^8^ but did not affect itaconate levels (Extended Data Fig. 3n). In summary, LPS drives a number of metabolic alterations, notably the generation of succinate from elevated glutaminolysis and an accumulation of itaconate.

**Extended Data Fig. 4:**
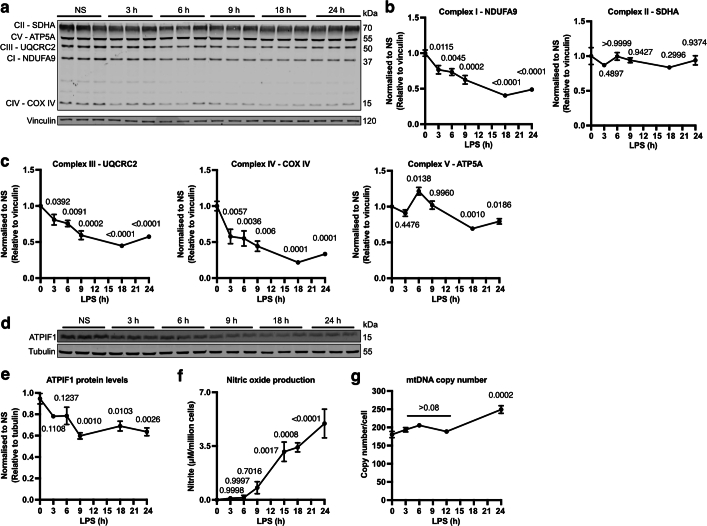
The electron transport chain and F_1_F_O_-ATP synthase in LPS-stimulated BMDMs. **a-c**, Western blot and quantification of complexes I-IV, F_1_F_O_-ATP synthase and vinculin loading control in BMDMs treated with LPS for 3–24 h or nonstimulated (NS) (n = 3) (Complex I 18 h, P = 3×10^-6^; 24 h, P = 1×10^-5^; Complex III 18 h, P = 6×10^-6^; 24 h, P = 9×10^-5^). Blots show three biological replicates for each condition and data are mean normalized to NS and vinculin loading control +/- S.E.M. of biological replicates. **d,e**, Western blot and quantification of ATPIF1 and tubulin in BMDMs treated with LPS for 3–24 h or NS (n = 3). Blot shows three biological replicates for each condition and data are mean normalized to NS and tubulin loading control. **f**, Nitrite levels in BMDMs stimulated with LPS for 3-6 h or NS (n = 3). Data are mean +/- S.E.M. of biological replicates. **g**, Mitochondrial DNA copy number measured by ddPCR (n = 3). Data are mean +/- S.E.M. of biological replicates. *P* values displayed above graphs calculated using one-way ANOVA for multiple comparisons. Source data

We then examined the expression of the electron transport chain and oxidative phosphorylation complexes, which showed that complexes I, III and IV decreased over 24 h of LPS treatment, whereas succinate dehydrogenase (SDH) subunit A remained stable following LPS treatment (Extended Data Fig. 4a–c). F_1_F_O_-ATP synthase remained stable until 9 h at which point it decreased (Extended Data Fig. 4a,c). This was confirmed by assessing ATP synthase puncta per mitochondrial volume after 24 h LPS treatment (Extended Data Fig. 2d). There was also a decrease in ATP synthase inhibitory factor 1 (ATPIF1) following LPS treatment (Extended Data Fig. 4d,e). Nitric oxide (NO) produced by iNOS, which has been shown to reduce complex I, II and III protein levels and activity in pro-inflammatory BMDMs^19^, was assessed as extracellular NO_2_^–^ and increased from 9 h LPS treatment (Extended Data Fig. 4f). In parallel, mitochondrial DNA copy number remained stable until 24 h LPS at which point there was a small increase (Extended Data Fig. 4g). Thus, the changes in mitochondrial superoxide and morphology occur in parallel with a metabolic shift and altered mitochondrial function.

### An elevated membrane potential drives superoxide production

**Extended Data Fig. 5:**
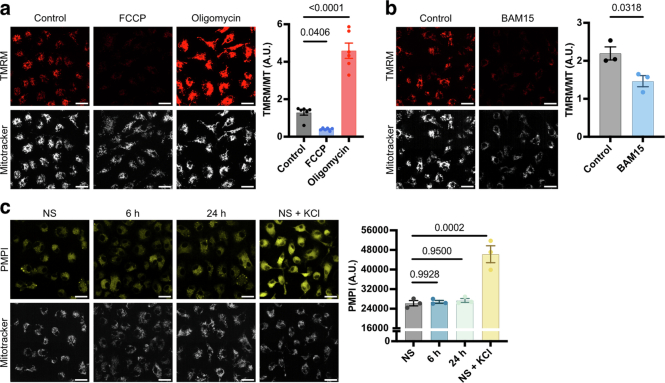
Measurement of mitochondrial membrane potential using TMRM in non-quenching mode. **a,b**, ∆ψ_m_ measured with TMRM normalized to Mitotracker Deep Red FM (Arbitrary Units (A.U.)) in nonstimulated (NS) BMDMs treated with 2 μM FCCP, 5 μM oligomycin or 10 μM BAM15 for 30 min from confocal live cell imaging (n = 6) (Oligomycin, P = 2×10^-7^). **c**, ∆ψ_p_ measured with plasma membrane potential indicator (PMPI) in NS BMDMs and in BMDMs stimulated with LPS for 6 h or 24 h and in NS BMDMs treated with 150 mM KCl from confocal live cell imaging (n = 3). Scale bars on all representative images are 20 μm. Data are mean +/- S.E.M. of biological replicates. *P* values displayed above graphs calculated using two-tailed Student’s *t*-test for paired comparisons or one-way ANOVA for multiple comparisons. Source data

The link between elevated mitochondrial superoxide production and metabolic reprogramming following LPS stimulation is not known. As Δp is a major determinant of mitochondrial superoxide production, we measured changes in Δp, as its dominant component Δψ_m_, by monitoring the Δψ_m_-dependent accumulation of the tetramethylrhodamine methyl ester (TMRM) probe in nonquenching mode using live-cell confocal microscopy^20^. The dynamic range of TMRM fluorescence was established from its maximum (+oligomycin) to its minimum (+uncouplers FCCP or BAM15) (Extended Data Fig. 5a,b). Δψ_m_ increased over time following LPS treatment reaching a maximum after 24 h LPS treatment (Fig. 1e,f). The uptake of TMRM into mitochondria can also be affected by its initial uptake across the plasma membrane in response to the plasma membrane potential (Δψ_p_). However, analysis of Δψ_p_^20,21^ in BMDMs treated with LPS showed that Δψ_p_ did not change (Extended Data Fig. 5c), indicating that our TMRM measurements are solely due to changes in Δψ_m_.

To determine how LPS induced an increase in Δψ_m_, we investigated the role of the F_1_F_O_-ATP synthase in altering Δψ_m_ following LPS stimulation because F_1_F_O_-ATP synthase either consumes or generates Δp. There was a large increase in Δψ_m_ when nonstimulated BMDMs were treated with oligomycin (Fig. 1e,g). Furthermore, this elevated Δp induced superoxide production in nonstimulated BMDMs (Fig. 1h,i) indicating that increased Δp alone could drive mitochondrial superoxide production.

Analysis of additional time points showed that acute treatment of oligomycin increased Δψ_m_ at early and late LPS treatment, but not between 6 and 9 h (Fig. 1e,g). Furthermore, oligomycin decreased the ATP/ADP ratio early and late but not from 6–15 h (Fig. 1j). Together, these data suggest that the cells switch from ATP production by oxidative phosphorylation to glycolysis, and then back again, resulting in reduced reliance on F_1_F_O_-ATP synthase activity, which drives an increase in Δp.

### CoQ pool reduction is necessary for superoxide production

**Fig. 2:**
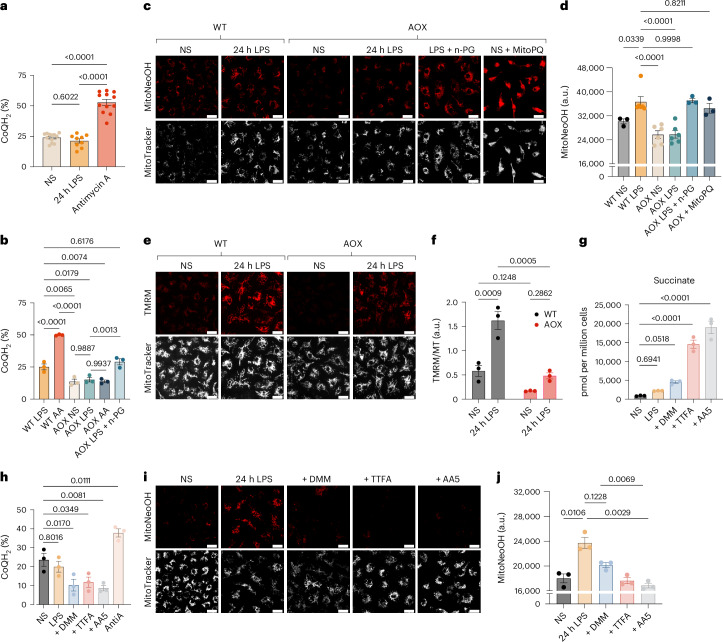
Reduction of the CoQ pool maintained by succinate oxidation is required for mitochondrial superoxide production. **a**,**b**, The redox state of the CoQ pool was measured by mass spectrometry in WT and AOX-expressing BMDMs that were NS, stimulated with LPS for 24 h, treated with 5 μM antimycin A for 15 min or stimulated with LPS and 100 μM n-PG for 24 h (*n* = 9 (24 h LPS), *n* = 12 (NS, antimycin A)) (NS versus antimycin A, *P* = 8 × 10^−12^; LPS versus antimycin A, *P* = 6 × 10^-12^; WT LPS versus WT AA, *P* = 5 × 10^-6^; WT AA versus AOX NS, *P* = 7 × 10^-8^). **c**,**d**, Mitochondrial superoxide production measured in NS WT BMDMs, WT BMDMs stimulated with LPS for 24 h and in AOX-expressing BMDMs that were NS, stimulated with LPS for 24 h with or without 100 μM n-PG or treated with 10 μM MitoPQ for 30 min from confocal live-cell imaging. MitoNeoOH fluorescence is measured as absolute intensity with arbitrary units (a.u.) (*n* = 3 (WT NS, AOX LPS + n-PG, AOX + MitoPQ) *n* = 6 (WT LPS, AOX NS, AOX LPS)) (WT LPS versus AOX NS *P* = 3 × 10^−5^; WT LPS versus AOX LPS *P* = 3 × 10^−5^). **e**,**f**, ∆ψ_m_ measured using TMRM normalized to MitoTracker Deep Red FM in WT and AOX-expressing BMDMs that were NS or stimulated with LPS for 24 h from confocal live-cell imaging (*n* = 3). **g**–**j**, NS BMDMs or BMDMs stimulated with LPS for 24 h and BMDMs stimulated with LPS for 12 h and then for a further 12 h with the addition of 10 mM DMM, 200 μM TTFA or 0.5 μM AA5. Succinate measured by mass spectrometry (*n* = 3) (NS versus TTFA, P = 3 × 10^−6^; NS versus AA5, *P* = 2 × 10^−7^) (**g**). The redox state of the CoQ pool measured by mass spectrometry (*n* = 3) (**h**). **i**,**j**, Mitochondrial superoxide production measured with MitoNeoD from confocal live-cell imaging. MitoNeoOH fluorescence is measured as absolute intensity (a.u.) (*n* = 3). Scale bars on all representative images, 20 μm. All data are mean ± s.e.m. of biological replicates. *P* values displayed above graphs calculated using one-way or two-way ANOVA. Source data

**Extended Data Fig. 6:**
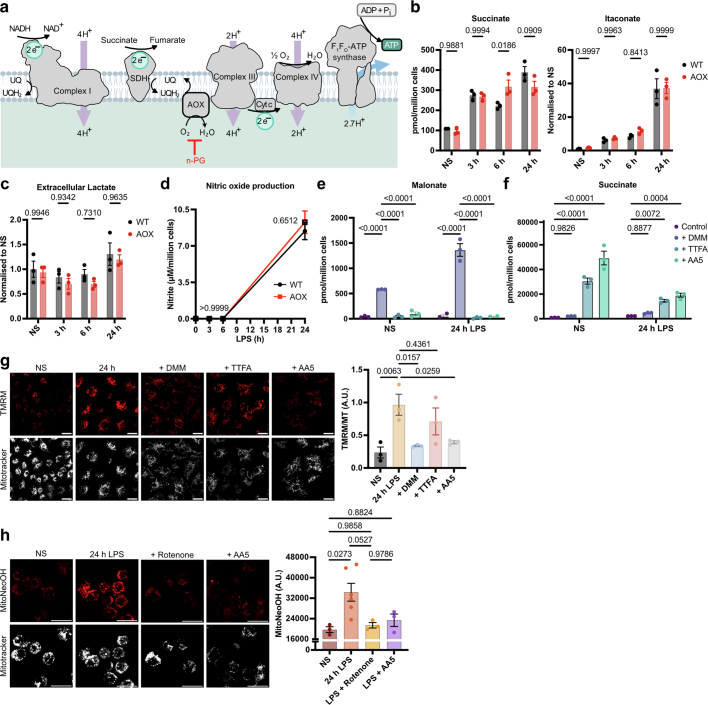
Ectopic expression of alternative oxidase in BMDMs. **a**, Transgenic expression of *Ciona intestinalis* alternative oxidase (AOX) in the mitochondrial inner membrane where it uses electrons from the CoQ pool to reduce oxygen to water without pumping protons or generating ROS. N-propyl gallate (n-PG) inhibits AOX. **b,c**, Succinate, itaconate and extracellular lactate measured by mass spectrometry in wild-type (WT) BMDMs and BMDMs expressing AOX that were nonstimulated (NS) or stimulated with LPS for 3 h, 6 h or 24 h (n = 3). **d**, Nitrite levels in WT BMDMs and BMDMs expressing AOX that were NS or stimulated with LPS for 3 h, 6 h or 24 h (n = 3). **e,f**, Malonate and succinate in BMDMs that were NS or stimulated with LPS for 12 h and then for a further 12 h with the addition of 10 mM dimethyl malonate (DMM), 200 μM thenoyltrifluoroacetone (TTFA) or 0.5 μM atpenin A5 (AA5) (n = 3). P values for malonate (NS DMM vs Control, P = 3×10^-6^; vs TTFA, P = 4×10^-6^; vs AA5, P = 1×10^-5^; LPS DMM vs Control, P = 1x-11; vs TTFA, P = 8×10-12; vs AA5, P = 8×10^-12^). P values for succinate (NS Control vs TTFA, P = 5×10^-7^; vs AA5, 4×10^-10^). **g**, ∆ψ_m_ measured by TMRM normalized to MitoTracker Deep Red FM with Arbitrary Units (A.U.) from confocal live cell imaging in BMDMs stimulated with LPS for 12 h and then for a further 12 h with the addition of 10 mM DMM, 200 μM TTFA or 0.5 μM AA5 (n = 3). Scale bars are 20 μm for all confocal images. **h**, THP-1 cells were differentiated to macrophage-like cells and treated with either 0.1 μM rotenone or 0.1 μM AA5 in the last 15 min of LPS treatment (n = 3 (NS, LPS + Rotenone, LPS + AA5), n = 6 (24 h LPS)). Mitochondrial superoxide production measured with MitoNeoD from confocal live cell imaging. Fluorescence intensity of MitoNeoOH is measured as absolute intensity (A.U.). Scale bars on all representative images are 20 μm. Data are mean +/- S.E.M. of biological replicates. *P* values displayed above graphs were calculated using two-tailed Student’s *t*-test for paired comparisons or one-way or two-way ANOVA for multiple comparisons. Source data

Superoxide production by the respiratory chain requires a reduced CoQ pool (more negative E_h_) so we measured the redox state of the CoQ pool in LPS-treated BMDMs by mass spectrometry^22^. The mitochondrial CoQ redox state did not change upon LPS treatment, remaining about half of the maximum obtained upon treatment with the complex III inhibitor antimycin A (Fig. 2a). To test how an oxidized CoQ pool would affect LPS-induced mitochondrial superoxide production, we used BMDMs expressing the alternative oxidase (AOX), which oxidizes the CoQ pool (Extended Data Fig. 6a)^23^. The CoQ pool of BMDMs expressing AOX was oxidized even following the addition of antimycin A and was restored by the AOX inhibitor n-propyl gallate (n-PG) (Fig. 2b). AOX decreased CoQ reduction in the presence of LPS (Fig. 2b) blocking LPS-induced superoxide production (Fig. 2c,d). Inhibition of AOX with n-PG restored mitochondrial superoxide production to similar levels induced by MitoPQ treatment (Fig. 2c,d). AOX-expressing BMDMs undergo comparable LPS-stimulated accumulation of succinate, itaconate and extracellular lactate (Extended Data Fig. 6b,c) and NO production (Extended Data Fig. 6d). AOX expression limited the LPS-induced increase in Δψ_m_ indicating that maintenance of a reduced CoQ pool promotes LPS-induced elevation of Δψ_m_ (Fig. 2e,f). Thus, increased mtROS production upon LPS stimulation is associated with an elevated Δp but not with any change to the CoQ pool redox state. However, reduction of the CoQ pool must be sustained for LPS-induced mitochondrial superoxide generation.

As maintenance of a reduced CoQ pool is necessary for mitochondrial superoxide production, in part to sustain Δp, we set out to determine the electron donors that maintain the CoQ redox state. Succinate accumulates following LPS stimulation, via enhanced glutaminolysis and has been implicated in promoting LPS-induced mtROS production^4^. Therefore, we next explored the effect of inhibiting SDH with dimethyl malonate (DMM), thenoyltrifluoroacetone (TTFA) or atpenin A5 (AA5) to block the supply of electrons from succinate to the CoQ pool. AA5 and TTFA occupy the CoQ binding site of complex II thus inhibiting CoQ reduction, while AA5 also partially inhibits oxidation of succinate^24,25^. In contrast, DMM is a prodrug that diffuses across membranes, is hydrolysed by esterases^26^ and increases cellular malonate levels (Extended Data Fig. 6e), which competitively inhibits SDH. BMDMs were treated with LPS for 12 h and then for a further 12 h with the addition of SDH inhibitors. Succinate levels continued to accumulate in the presence of SDH inhibitors (Fig. 2g and Extended Data Fig. 6f). Thus, although there are high levels of succinate present during LPS treatment (Fig. 1c), we have demonstrated that succinate is still oxidized at this late stage (Fig. 2g). Inhibition of succinate oxidation resulted in an oxidized CoQ pool (Fig. 2h), abrogated the LPS-induced increase in Δψ_m_ (Extended Data Fig. 6g), and inhibited mitochondrial superoxide production following LPS treatment (Fig. 2i,j). Thus, succinate oxidation is necessary to both elevate Δψ_m_ and maintain reduction of the CoQ pool to drive superoxide production. However, there are many other electron donors that may also contribute to the maintenance of reduced CoQ. Indeed, it has been shown that knockout of mitochondrial glycerol 3-phosphate dehydrogenase (GPD2) decreases mtROS production in pro-inflammatory macrophages^27^. Having said this, the substantial effects of inhibiting SDH on the CoQ redox state and mtROS generation (Fig. 2h–j) suggest that succinate oxidation dominates this process.

We then examined whether human macrophages produce mtROS by the same mechanism. We differentiated the human monocytic cell line THP-1 to macrophage-like cells using phorbol-12-myristate-13-acetate (PMA) and measured mitochondrial superoxide production using MitoNeoD. Mitochondrial superoxide was generated by these cells following LPS stimulation and its production was sensitive to rotenone treatment and inhibited by the SDH inhibitor AA5 (Extended Data Fig. 6h). This indicates that similar to murine BMDMs, LPS-stimulated human macrophages not only generate mtROS but its production is complex I dependent and requires succinate oxidation.

### LPS-induced superoxide is produced by RET at complex I

**Fig. 3:**
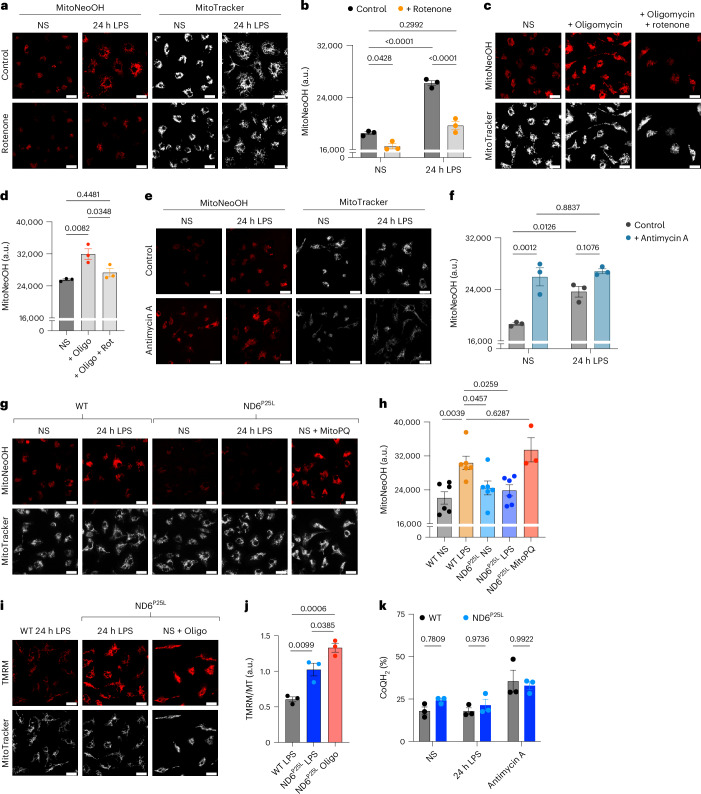
LPS-induced mitochondrial superoxide is produced by reverse electron transport at complex I. **a**–**h**, Mitochondrial superoxide production measured with MitoNeoD from confocal live-cell imaging. Fluorescence intensity of MitoNeoOH is measured as absolute intensity with arbitrary units (a.u.). BMDMs stimulated with LPS for 24 h were treated with 0.5 μM rotenone in the last 30 min of LPS treatment or for 30 min with 0.5 μM rotenone or 5 μM oligomycin in NS cells (*n* = 3) (NS versus LPS, *P* = 7 × 10^−6^; LPS versus LPS + rotenone, *P* = 2 × 10^−5^) (**a**–**d**). BMDMs stimulated with LPS for 24 h were treated with 5 μM antimycin A in the last 30 min of LPS treatment or for 30 min in NS cells (*n* = 3) (**e**,**f**). BMDMs from WT and ND6 ^P25L^ mice stimulated with LPS for 24 h, NS or treated with 5 μM MitoPQ for 30 min (*n* = 3 (ND6^P25L^ MitoPQ), *n* = 6 (WT NS, WT LPS, ND6^P25L^ NS, ND6^P25L^ LPS)) (**g**,**h**). **i**,**j**, ∆ψ_m_ measured using TMRM normalized to MitoTracker Deep Red FM in BMDMs from WT and ND6^P25L^ mice that were treated with 5 μM oligomycin for 30 min or stimulated with LPS for 24 h from confocal live-cell imaging (*n* = 3). Scale bars on all representative images, 20 μm. **k**, The redox state of the CoQ pool measured by mass spectrometry in BMDMs from WT and ND6^P25L^ mice that were NS, stimulated with LPS for 24 h or treated with 5 μM antimycin A for 15 min (*n* = 3). All data are mean ± s.e.m. of biological replicates. *P* values displayed above graphs calculated using two-tailed Student’s *t*-test for paired comparisons or one-way or two-way ANOVA for multiple comparisons. Source data

**Extended Data Fig. 7:**
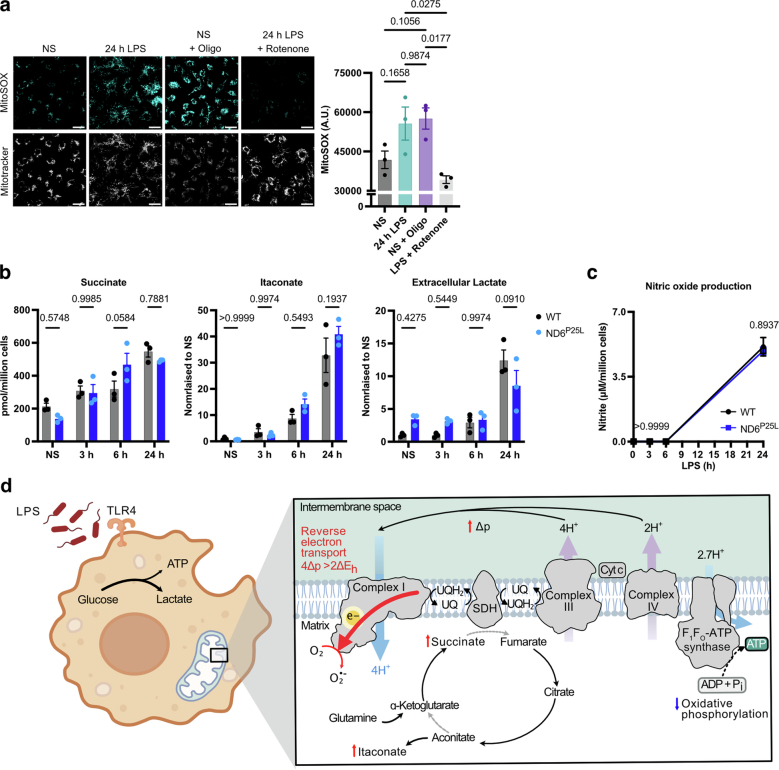
LPS-induced mitochondrial superoxide production and metabolic reprogramming in BMDMs from ND6^P25L^ mice. **a**, Mitochondrial superoxide production measured with MitoSOX from confocal live cell imaging in nonstimulated (NS) BMDMs, BMDMs stimulated with LPS for 24 h, NS BMDMs treated with 5 μM oligomycin and BMDMs stimulated with LPS for 24 h and treated with 0.5 μM rotenone in the last 30 min of the LPS treatment (n = 3). Fluorescence intensity is measured as absolute intensity with Arbitrary Units (A.U.) (n = 3). Scale bars on all representative images are 20 μm. *P* values displayed above graphs calculated using one-way ANOVA. **b**, Succinate, itaconate and extracellular lactate measured by mass spectrometry in BMDMs from wild-type (WT) and ND6^P25L^ mice that were NS or stimulated with LPS for 3 h, 6 h or 24 h (n = 3). **c**, Nitrite levels in BMDMs from WT and ND6^P25L^ mice that were NS or stimulated with LPS for 3 h, 6 h or 24 h (n = 3). All data are mean +/- S.E.M. of biological replicates. *P* values displayed above graphs calculated using two-tailed Student’s *t*-test for paired comparisons or one-way ANOVA for multiple comparisons. **d**, LPS stimulation induces metabolic reprogramming consisting of a switch from ATP production by oxidative phosphorylation to glycolysis. The abundance of tricarboxylic acid cycle metabolites is also altered, including an accumulation of succinate and itaconate. These changes result in an elevated ∆p and a reduced CoQ pool which drives superoxide production by RET at complex I. LPS, lipopolysaccharide; TLR4, Toll-like receptor-4; SDH, succinate dehydrogenase; ∆p, proton motive force; ∆E_h_, difference in reduction potential between NAD/NADH and UQ/UQH_2_ couples. Source data

Mitochondrial superoxide can be produced by various means along the ETC: by FET, RET and at complex III. The increase in MitoNeoOH and MitoSOX fluorescence after LPS treatment was abolished by rotenone (Fig. 3a,b and Extended Data Fig. 7a), suggesting that mitochondrial superoxide production is dependent on RET at complex I. We also showed that oligomycin induced superoxide production in nonstimulated BMDMs was sensitive to rotenone (Fig. 3c,d). However, rotenone treatment may also affect superoxide production at complex III by blocking electron flow to the CoQ pool. Superoxide can be produced at complex III via donation of an electron from ubisemiquinone to oxygen rather than to cytochrome b_l_ (ref. ^6^). Antimycin A binds to the Q_i_ site of complex III and prevents movement of electrons by the b cytochromes, which enhances the lifetime of ubisemiquinone and promotes superoxide production^6,28^. Thus, antimycin A treatment induced mitochondrial superoxide production in nonstimulated BMDMs (Fig. 3e,f). The ubisemiquinone could be stabilized by a high Δp and succinate oxidation^6^. Therefore, mtROS produced following LPS treatment is either produced at complex III or at complex I by RET^6^.

To distinguish between ROS generated at complex III and ROS produced by RET at complex I, we used a genetic model that is incapable of producing superoxide by RET. The *ND6 G14600A* mitochondrial DNA (mtDNA) mutation results in a P25L substitution in the ND6 subunit of complex I, which prevents RET but does not affect FET or complex III (refs. ^29–32^). BMDMs from ND6^P25L^ mice did not produce superoxide upon LPS treatment but MitoPQ did (Fig. 3g,h). Succinate, itaconate and extracellular lactate levels were not affected in LPS-stimulated BMDMs from ND6^P25L^ mice (Extended Data Fig. 7b) nor was NO production (Extended Data Fig. 7c). The Δψ_m_ following 24 h LPS treatment was higher in BMDMs from ND6^P25L^ mice than from wild-type mice and was increased further with oligomycin treatment (Fig. 3i,j), whereas the CoQ redox state of BMDMs from both wild-type and ND6^P25L^ mice was comparable (Fig. 3k). These data demonstrate that LPS-activated macrophages produce superoxide by RET at complex I, which in turn is driven by an elevated Δψ_m_ and maintenance of a reduced CoQ pool in agreement with the requirement of 4Δp > 2ΔE_h_ for RET (Extended Data Fig. 7d).

### Regulation of LPS-induced cytokine production by mtROS

**Extended Data Fig. 8:**
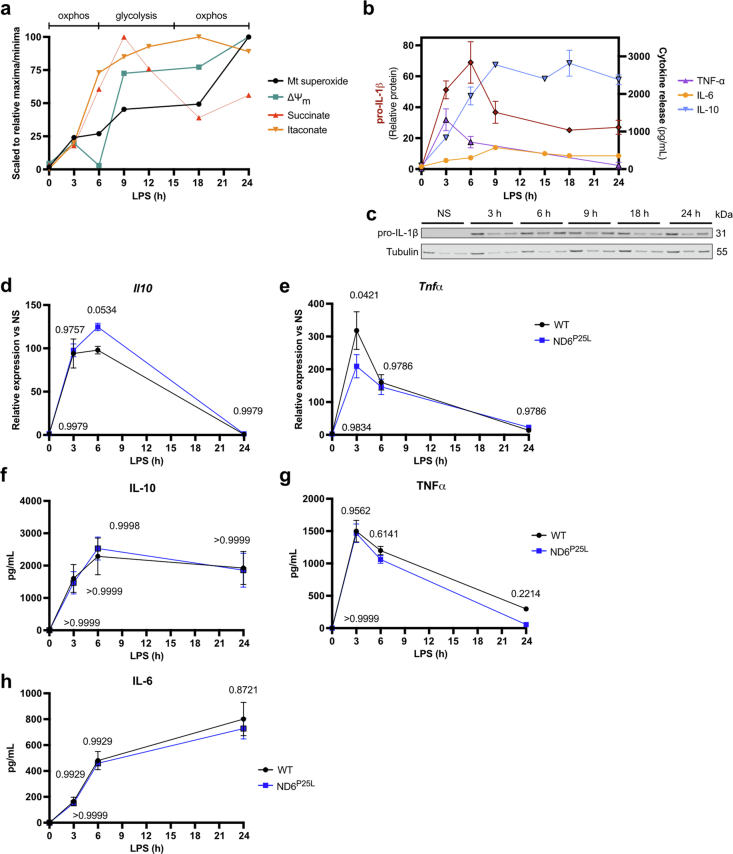
Regulation of LPS-induced cytokine production by mtROS in BMDMs. **a**, Mitochondrial superoxide production measured with MitoNeoD, ∆ψ_m_ measured with TMRM, and succinate and itaconate measured by mass spectrometry in nonstimulated (NS) BMDMs or BMDMs stimulated with LPS for 3–24 h (n = 6-9). Data are scaled to the relative minima and maxima of each measurement (MitoNeoD (n = 3 (NS, 6 h, 9 h), n = 6 (3 h, 18 h, 24 h)); ∆ψ_m_ (n = 6 (3 h, 6 h, 18 h, 24 h), n = 9 (NS, 9 h)); succinate (n = 3); itaconate (n = 3)). **b**, IL-10, TNFα and IL-6 release measured by ELISA from NS BMDMs or BMDMs stimulated with LPS for 3–24 h (n = 3). Data are mean +/- S.E.M. of biological replicates. **b,c**, Western blot and quantification of pro-IL-1β and tubulin (n = 3). Blot shows three biological replicates for each condition and data are mean normalized to NS and tubulin loading control +/- S.E.M. of biological replicates. **d-h**, BMDMs from wild-type (WT) and ND6^P25L^ mice that were NS or stimulated with LPS for 3 h, 6 h or 24 h LPS (n = 3). **d, e**, *Il10* and *TNFα* expression assessed by RT–qPCR (n = 3). **f-h**, IL-10, TNFα and IL-6 release measured by ELISA (n = 3). Data are mean +/- S.E.M. of biological replicates. *P* values displayed above graphs calculated using two-tailed Student’s *t*-test for paired comparisons. Source data

The relative time courses of the changes in mitochondrial superoxide, Δψ_m_, succinate and itaconate are shown in Extended Data Fig. 8a. These changes occurred in tandem with cytokine production (Extended Data Fig. 8b,c), which is strictly regulated by auto-regulation through feedback loops and regulation by mitochondria-derived signals^33^. The pro-inflammatory cytokine TNF was rapidly produced within 3 h of TLR4 activation, while pro-interleukin (IL)-1β and IL-6 peaked at 6 h and 9 h, respectively (Extended Data Fig. 8b,c). These pro-inflammatory cytokines are negatively regulated by IL-10, which was produced within 3 h of LPS stimulation (Extended Data Fig. 8b), and acts as an autocrine signal via the IL-10 receptor (CD210; IL-10R)^34–36^.

**Extended Data Fig. 9:**
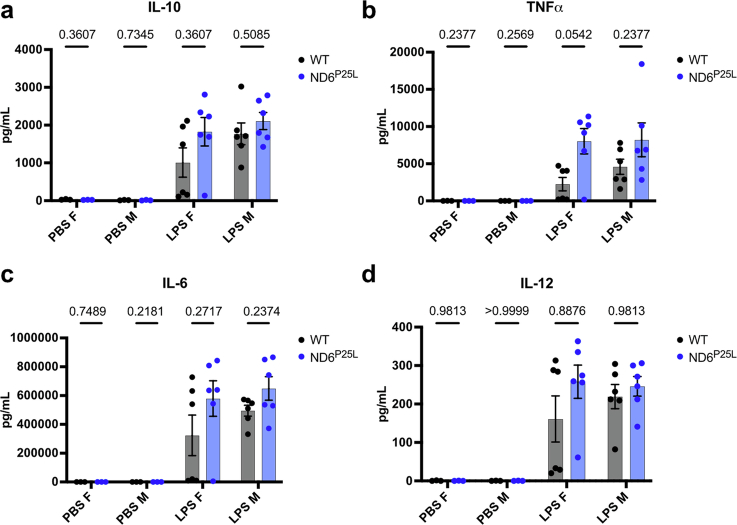
Serum cytokine levels in an *in vivo* model of sepsis from wild-type and ND6^P25L^ mice. **a–d**, Serum cytokines measured in female (F) and male (M) wild-type (WT) and ND6^P25L^ mice 2 h post intraperitoneal injection of PBS or 2.5 mg/kg LPS (n = 3 (PBS female and PBS male) and (n = 6 (LPS female and LPS male)). Data are mean +/- S.E.M. of biological replicates. *P* values displayed above graphs calculated using two-tailed Student’s *t*-test for paired comparisons. Source data

To explore the role of mtROS in cytokine regulation, we assessed cytokine release in BMDMs from wild-type and ND6^P25L^ mice. *Il10* expression was higher at 6 h LPS treatment in BMDMs from ND6^P25L^ mice, whereas *Tnf* expression was lower at 3 h (Extended Data Fig. 8d,e). However, IL-10 and TNF release were the same from BMDMs from wild-type and ND6^P25L^ mice (Extended Data Fig. 8f,g), as was IL-6 release (Extended Data Fig. 8h). These results suggest that the inability to produce mtROS by RET at complex I does not affect LPS-induced cytokine production in BMDMs. We also assessed cytokine production in a murine in vivo model of sepsis. Serum cytokine levels were the same in wild-type and ND6^P25L^ mice following 2 h LPS treatment (Extended Data Fig. 9a-d). Thus, using a genetic model incapable of producing mtROS by RET, we were able to disentangle the effects of mitochondria-derived metabolites, such as succinate, and mtROS in the regulation of LPS-induced cytokine production to show that mtROS does not substantially contribute to the regulation of cytokine generation in LPS-stimulated macrophages.

### RET-ROS regulates IL-1β release

MtROS have also been implicated in the regulation of IL-1β release and NLRP3 (NOD-, LRR-, and pyrin domain-containing protein 3) inflammasome activation^37–43^. NLRP3 inflammasome activation requires two signals: initial priming by LPS induces expression of pro-IL-1β and NLRP3 inflammasome components, which are then activated by a second signal leading to caspase-1 cleavage of pro-IL-1β and gasdermin D (GSDMD) resulting in pyroptosis and release of mature IL-1β^44,45^. Following initial priming, inflammasome activation can be triggered by highly diverse molecules such as ATP, pore-forming toxins and particulates^44,45^. The ability of such diverse stimuli to bring about inflammasome activation is puzzling and there have been suggestions that these signals may converge on a mitochondrial pathway mediated via mtROS^37,46^; however, others propose that while the mitochondrial electron transport chain is essential in NLRP3 inflammasome activation, activation is independent of mtROS and instead is triggered by phosphocreatine dependent ATP production^47^. Thus, the role of mtROS in inflammasome activation remains unclear.

**Fig. 4:**
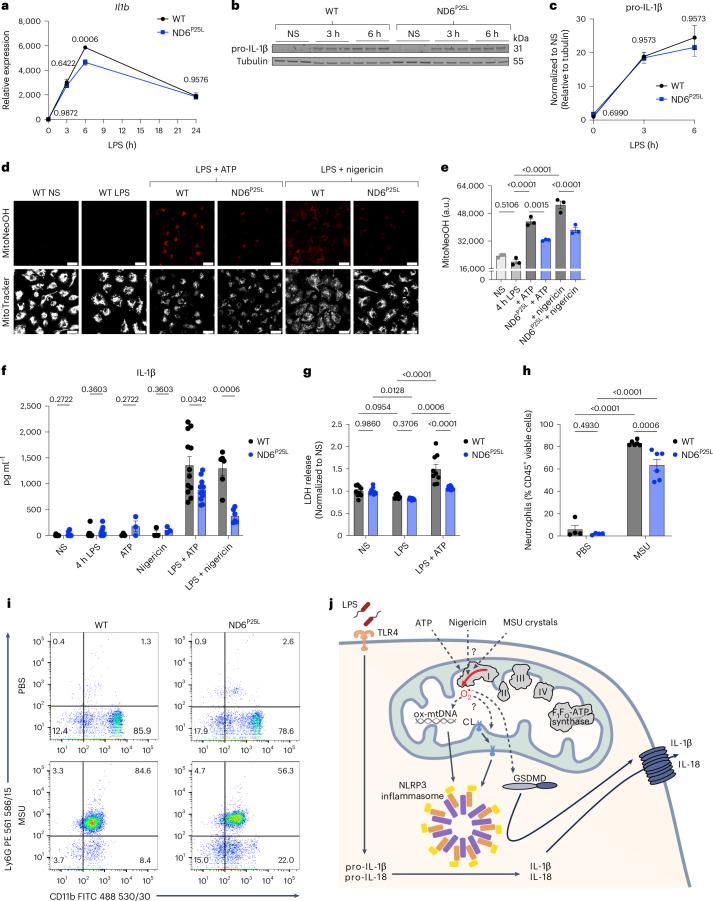
MtROS production regulates IL-1β release during NLRP3 inflammasome activation. **a**, *Il1β* expression in BMDMs from WT and ND6^P25L^ mice stimulated with LPS for 3 h, 6 h or 24 h or NS (*n* = 3). **b**,**c**, Western blot and quantification of pro-IL-1β and tubulin in BMDMs from WT and ND6^P25L^ mice stimulated with LPS for 3 h or 6 h or NS (*n* = 3). Blot shows three biological replicates for each condition and data are mean normalized to NS and tubulin loading control ± s.e.m. of biological replicates. **d**–**g**, BMDMs from WT and ND6^P25L^ mice that were NS, stimulated with LPS for 4 h or primed with LPS for 4 h followed by treatment with 5 mM ATP or 15 μM nigericin for 1 h. Mitochondrial superoxide production measured with MitoNeoD (**d**,**e**). BMDMs were incubated with MitoNeoD in the last 30 min of LPS priming and imaged within 5 min of ATP or nigericin addition. MitoNeoOH fluorescence is measured as absolute intensity with arbitrary units (a.u.) (*n* = 3) (LPS versus ATP, *P* = 6 × 10^−7^; LPS versus nigericin, *P* = 1 × 10^−8^; nigericin versus ND6^P25L^ + nigericin, *P* = 1 × 10^−4^). Scale bars on all representative images, 20 μm. IL-1β release (*n* = 3 (ATP, nigericin), *n* = 6 (LPS + nigericin), *n* = 9 (NS, 4 h LPS), *n* = 12 (LPS + ATP)) (**f**). LDH release (*n* = 9) (WT LPS versus ATP, *P* = 7 × 10^−12^; WT LPS + ATP versus ND6^P25L^ LPS + ATP, 1 × 10^−7^) (**g**). **h**,**i**, WT and ND6^P25L^ mice were injected intraperitoneally with PBS (*n* = 4) or MSU crystals (30 mg kg^−1^, *n* = 6) for 6 h (WT PBS versus MSU, *P* = 8 × 10^−11^; ND6^P25L^ PBS versus MSU, 2 × 10^−9^). Neutrophils were identified as CD45^+^CD11b^+^Ly6G^+^ cells. Data are displayed as representative dot plots and neutrophils as a percentage of CD45^+^ live cells in each condition. **a**,**c**,**e**–**h**, All data are mean ± s.e.m. of biological replicates. *P* values displayed above graphs calculated using two-tailed Student’s *t*-test for paired comparisons or one-way ANOVA or two-way ANOVA for multiple comparisons. **j**, The role of mtROS in the regulation of NLRP3 inflammasome activation and IL-1β release. CL, cardiolipin; ox-mtDNA, oxidized mtDNA. Source data

**Extended Data Fig. 10:**
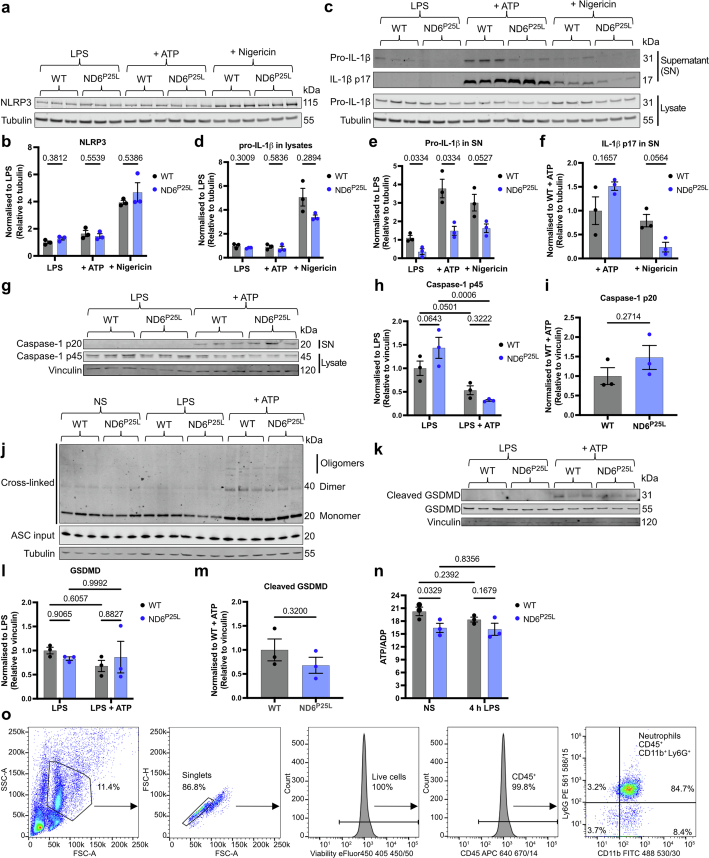
The role of mtROS production by RET in NLRP3 inflammasome activation. BMDMs from wild-type (WT) and ND6^P25L^ mice stimulated with LPS for 4 h or primed with LPS for 4 h followed by treatment with 5 mM ATP or 15 μM nigericin for 1 h. **a,b**, Western blot and quantification of NLRP3 and tubulin (n = 3). Blots show three biological replicates for each condition and data are mean normalized to NS and tubulin loading control +/- S.E.M. of biological replicates. **c-f**, Western blot and quantification of pro- and mature IL-1β and tubulin in cell lysates and protein released into cell supernatant (n = 3). Blots show three biological replicates for each condition and data are mean normalized to NS and tubulin loading control +/- S.E.M. of biological replicates. **g-i**, Western blot and quantification of caspase-1 p45 and vinculin in cell lysates and caspase-1 p20 released into cell supernatant (n = 3). Blots show three biological replicates for each condition and data are mean normalized to NS and vinculin loading control +/- S.E.M. of biological replicates. **j**, Western blot of ASC protein with or without treatment with cross-linking agent BS3 (n = 3). Blot shows three biological replicates for each condition. **k-m**, Western blot and quantification of intact and cleaved gasdermin D (GSDMD) (n = 3). Blots show three biological replicates for each condition and data are mean normalized to NS and vinculin loading control +/- S.E.M. of biological replicates. **n**, The ATP/ADP ratio measured per million cells in nonstimulated (NS) BMDMs and BMDMs stimulated with LPS for 4 h from WT and ND6^P25L^ mice (n = 3). Data are mean +/- S.E.M. of biological replicates. *P* values displayed above graphs calculated using two-tailed Student’s *t*-test for paired comparisons or one-way or two-way ANOVA for multiple comparisons. **o**, Gating strategy for neutrophils as analysed by flow cytometry. Source data

To address this question, we assessed IL-1β production in BMDMs from ND6^P25L^ mice. *Il1b* transcription was decreased at 6 h LPS treatment in ND6^P25L^ mutant BMDMs (Fig. 4a); however, pro-IL-1β and NLRP3 levels in BMDMs from ND6^P25L^ mice were comparable with wild-type (Fig. 4b,c and Extended Data Fig. 10a,b). This shows that mtROS does not impact pro-IL-1β production or NLRP3 expression during LPS priming. Therefore, we next investigated mtROS production during NLRP3 inflammasome activation. MitoNeoOH fluorescence greatly increased with ATP and nigericin treatment of wild-type BMDMs primed with LPS for 4 h, whereas LPS-primed BMDMs from ND6^P25L^ mice did not produce mtROS following ATP or nigericin treatment (Fig. 4d,e) demonstrating that mtROS is generated by RET during NLRP3 inflammasome activation.

We next measured how mtROS generation by RET affects the activation of the NLRP3 inflammasome. While pro-IL-1β expression was not affected in BMDMs from ND6^P25L^ mice, release of pro-IL-1β was decreased in LPS-primed BMDMs from ND6^P25L^ mice following ATP or nigericin treatment (Extended Data Fig. 10c–e). Furthermore, the release of mature IL-1β following NLRP3 inflammasome activation was reduced from BMDMs from ND6^P25L^ mice (Fig. 4f and Extended Data Fig. 8c,f). Thus, we have demonstrated that mtROS production by RET regulates IL-1β release following NLRP3 inflammasome activation.

Another consequence of NLRP3 inflammasome activation is pyroptosis mediated via GSDMD oligomerization and pore formation^44^. Lactate dehydrogenase (LDH) release, used as a measure of pyroptosis, was lower from BMDMs from ND6^P25L^ mice primed with LPS and treated with ATP (Fig. 4g). Therefore, building on other studies that mtROS regulates pyroptosis^43,48,49^, we further show that pyroptosis is regulated by mtROS produced by RET.

To determine whether mtROS regulates NLRP3 inflammasome assembly we measured the formation of ASC multimeric complexes and procaspase-1 cleavage. ASC is an adaptor protein, which is recruited by NLRP3 to form multimeric complexes called ASC specks and subsequently recruits procaspase-1 via CARD-CARD domains resulting in caspase-1 self-cleavage^44^. Both the formation of ASC specks and caspase-1 cleavage, determined by western blotting, were comparable in wild-type and ND6^P25L^ BMDMs (Extended Data Fig. 10g–j). This suggests that regulation by mtROS does not affect NLRP3 inflammasome assembly but instead acts downstream via regulation of GSDMD pore formation. While there was no change in GSDMD expression or cleavage in BMDMs from ND6^P25L^ mice compared with wild-type (Extended Data Fig. 10k–m), the impaired release of IL-1β (Fig. 4f and Extended Data Fig. 10c,e,f) and abrogated cell death (Fig. 4g) measured in BMDMs from ND6^P25L^ mice suggests that GSDMD pore formation is regulated by RET-ROS. This is consistent with recent reports that have shown that GSDMD oligomerization and pore formation is regulated by ROS-mediated oxidation of redox-sensitive cysteine residues^43,48,49^.

These findings contrast with a report that NLRP3 inflammasome activation is independent of mtROS and instead is triggered by phosphocreatine dependent ATP production^47^. However, the ATP/ADP levels were comparable in BMDMs from wild-type and ND6^P25L^ mice (Extended Data Fig. 10n). Thus our data support other studies that have implicated mtROS in NLRP3 inflammasome activation^38^.

In addition to the use of BMDMs, we further investigated the role of mtROS during NLRP3 inflammasome activation in vivo by intraperitoneal injection of monosodium urate (MSU) crystals. MSU crystals activate the NLRP3 inflammasome and are the causative agents of gout, a form of arthritis associated with oedema and erythema of the joints and severe pain, conditions associated with strong neutrophil recruitment to the intra-articular and periarticular spaces^50^. A murine MSU-induced peritonitis model showed that neutrophil infiltration to the peritoneal cavity was reduced in ND6^P25L^ mice (Fig. 4h,i and Extended Data Fig. 10o) and we also observed a reduced pain response to MSU crystal injection in ND6^P25L^ mice (observation unquantified). Thus, we demonstrated that mtROS production by RET is important in the regulation of NLRP3 inflammasome activation in vivo.

## Discussion

Overall, we have outlined a detailed temporal analysis of metabolic reprogramming following LPS stimulation to elucidate the driving forces behind mtROS production and thus we have clarified that in LPS-stimulated macrophages mtROS is generated by RET at complex I. Furthermore, using the ND6^P25L^ mouse model, which is not capable of producing mtROS by RET, we were able to disentangle the signalling roles of mtROS and metabolites in the regulation of macrophage cytokine production to demonstrate that mtROS produced by RET regulates IL-1β release following NLRP3 inflammasome activation.

The mechanism by which mtROS production by RET regulates IL-1β release following NLRP3 inflammasome activation was beyond the scope of this paper but our results suggest that mtROS production by RET regulates GSDMD pore formation in agreement with recently published results demonstrating that GSDMD oxidation affects its ability to oligomerize and form a pore^43,48^. However, oxidation of cardiolipin^51,52^ and mtDNA^40,42,53^ have also been implicated in NLRP3 inflammasome activation downstream of ROS production (Fig. 4j). Furthermore, our results do not exclude a role for other redox signals in the regulation of inflammasome activation. Indeed, it has been shown that itaconylation and NO play a role in inflammasome activation in macrophages primed with LPS for 12 h, referred to as tolerized macrophages^54^. Although itaconate and NO in BMDMs from ND6^P25L^ mice were comparable with wild-type levels, we only investigated the classical inflammasome model by priming BMDMs with LPS for 4 h (refs. ^55,56^). Therefore, it is possible that different redox signals regulate inflammasome activation in distinct ways with inflammasome activation in tolerized macrophages regulated by itaconylation and NO, while classical inflammasome activation is regulated by mtROS^38,43,54,57,58^.

Collectively, our findings have clarified the mechanism of mtROS production in pro-inflammatory macrophages and the influence of mtROS on macrophage cytokine production, while highlighting the dynamic nature of LPS-induced metabolic reprogramming. Moreover, the tools and genetic model used in this paper will be valuable in further elucidating the role of mitochondrial function and mtROS in the immune system.

## Methods

### Animals

All mouse experiments were carried out in accordance with the UK Animals (Scientific Procedures) Act, 1986 (Home Office PPL number P6C97520A and PP1740969). All procedures were approved by the University of Cambridge Animal Welfare and Ethical Review Body Committee. Wild-type (WT) mice (C57BL/6J) were purchased from Charles River Laboratories. The ND6^P25L^ mouse strain^30^ was generously provided by D. Wallace, University of Pennsylvania and backcrossed onto the C57BL/6J background. C57Bl/6J mice carrying a single copy of *Ciona* *intestinalis* AOX gene in the Rosa26 locus were generated by T. Braun, H. T. Jacobs and M. Szibor^23^. Mice were bred and maintained in pathogen-free facilities with a 12-h light–dark cycle, a room temperature of 19–22 °C, relative humidity 55 ± 10% and with ad lib access to food and water.

### Reagents and compounds

LPS from *Escherichia* *coli*, serotype EH100 (Alexis) was stored at 1 mg ml^−1^ in phosphate-buffered saline (PBS) at 4 °C, sonicated for 5 min before use and used at a final concentration of 100 ng ml^−1^.

All compounds for cell treatments were used at the following final concentrations from stock solutions dissolved in the corresponding solvents stated here: rotenone (0.5 μM; dimethylsulfoxide (DMSO); Santa Cruz), antimycin A (5 μM; ethanol; Sigma), carbonyl cyanide-p-trifluoromethoxyphenylhydrazone (FCCP; 2 μM; ethanol; Sigma), BAM15 (10 μM; ethanol; Sigma), oligomycin A from *Streptomyces* *diastatochromogenes* (5 μM; ethanol; Sigma), n-propyl gallate (100 μM; ethanol; Sigma), DMM (10 mM; Fisher Scientific), AA5 (0.5 μM; DMSO; Abcam), TTFA (200 μM; DMSO; Sigma), BPTES (10 μM; DMSO; Sigma), ATP (5 mM; water; Sigma), nigericin (15 μM; DMSO; Sigma), KCl (150 mM; water; Fisher Scientific) and MitoPQ (10 μM; ethanol). MitoNeoD and MitoPQ were provided by R. C. Hartley.

### Antibodies

Working dilutions of antibodies were 1:1,000 unless otherwise stated. Anti-ASC (67824), ATPIF1 (8528), caspase-1 (E2Z1C) (24232), cleaved caspase-1 (Asp296) (89332), Cleaved gasdermin D (Asp276) (10137), Cleaved IL-1β (Asp117) (63124), IL-1β (12507), GAPDH (5174), gasdermin D (E9S1X) (39754), hexokinase I (2024T), NLRP3 (15101), PKM1/2 (3190), PKM2 (4053T), pyruvate dehydrogenase (3205) and VDAC (4661) antibodies were purchased from Cell Signaling. Anti-IRG1 (ab222411), ATP Synthase (MAB3494; 1:500 dilution) and TOMM20 (ab232589; 1:500 dilution) antibodies and OXPHOS blue native antibody cocktail (1:500 dilution; ab110412) were purchased from Abcam. Anti-tubulin (T9026) and vinculin (SAB4200729) antibodies were purchased from Sigma-Aldrich. Anti-TOMM20 (11802-1-AP) was purchased from Proteintech. Mouse IgG (926-68070) and rabbit IgG (926-3211) antibodies, both used at 1:15,000 dilution, were purchased from Li-cor and used for detection of all primary antibodies except for cleaved gasdermin D. Cleaved gasdermin D was detected using anti-rabbit IgG (W4011) purchased from Promega and analysed using Amersham ECL prime western blotting detection reagents (RPN2232; Cytiva).

Fluorescent antibodies A-21125 (Invitrogen), 4412S (Cell Signaling) and Alexa Fluor 568 rabbit IgG (A-11036; Thermo Fisher) were all used at 1:1,000 dilution.

### Generation of BMDMs

Male mice aged 10–18 weeks were killed by cervical dislocation and death was confirmed by exsanguination. Bone marrow was collected from the tibia and fibula. Cells were pelleted by centrifugation at 425*g* (1,500 rpm) for 5 min and red blood cells were lysed using a hypotonic red blood cell lysis buffer (Cambridge Bioscience). The remaining cells were pelleted by centrifugation at 425*g* (1,500 rpm) for 5 min and filtered through a 70-μm nylon mesh. Obtained monocytes were differentiated in DMEM containing 20% L929 supernatant, 10% foetal bovine serum (FBS) (Gibco) and 1% penicillin–streptomycin (pen–strep) (Gibco) for 3 days in 10-cm dishes, at which point, additional L929 supernatant (10%) was added and the cells were differentiated for a further 3 days. BMDMs were scraped, a sample was stained with Trypan blue, counted using an automated cell counter (Thermo Fisher) and then plated at 1 × 10^6^ cells per ml in DMEM containing 10% L929 supernatant, 10% FBS and 1% pen–strep. BMDMs were plated in six-well cell culture plates containing 1 × 10^6^ cells per well unless otherwise stated and left overnight to adhere.

### THP-1 cell culture and differentiation

Human monocytic THP-1 cells (ATCC) were cultured in RPMI 1640 Medium (Gibco) supplemented with 2 mM l-glutamine, 10% FBS and 1% pen–strep. THP-1 cells were differentiated to macrophage-like cells by treatment with 10 nM PMA (Thermo Fisher) for 24 h. These cells were rested for a further 48 h in RPMI without PMA before stimulation with 100 ng ml^−1^ LPS.

### Metabolomics

Succinate, itaconate, malonate and lactate were extracted and analysed as previously described^59^. Extracellular metabolites were extracted from 50 μl extracellular medium in 450 μl mass spectrometry (MS) extraction buffer (50% (v/v) methanol, 30% (v/v) acetonitrile and 20% (v/v) MS-grade water) supplemented with 1 nmol [^13^C_4_]-succinate and [^13^C_3_]-malonate. Cells were quickly washed in ice-cold PBS and metabolites were then extracted in 500 μl MS extraction buffer supplemented with 1 nmol of [^13^C_4_]-succinate and [^13^C_3_]-malonate. Succinate and malonate were quantified using the internal standards. Relative concentrations of itaconate and lactate were determined using the area ratio of the spectra.

Cells were extracted for untargeted metabolomics using the following method: cells were quickly washed in PBS and metabolites were then extracted in MS extraction buffer supplemented with 5 μM valine-d8 (CK isotopes, DLM-488) and 5 μM hippuric acid-d5 (CK isotopes, DLM-7703). Samples were placed in a pre-chilled Eppendorf tube and agitated by centrifugation at 17,000*g* for 15 min at 4 °C followed by incubation at −20 °C for 1 h and centrifugation at 17,000*g* at 4 °C for 10 min. The supernatants were transferred to MS vials for storage at −80 °C until analysis.

Untargeted metabolomics was performed using a Thermo Scientific Q Exactive Hybrid Quadrupole-Orbitrap mass spectrometer (HRMS) coupled to a Dionex Ultimate 3000 UHPLC^55^. The mass spectrometer was operated in full-scan, polarity-switching mode, with the spray voltage set to +4.5 kV/−3.5 kV, the heated capillary held at 280 °C and the heated electrospray ionization probe held at 320 °C. The sheath gas flow was set to 40 units, the auxiliary gas flow was set to 15 units and the sweep gas flow was set to 0 unit. HRMS data acquisition was performed in a range of *m/z* = 70–900, with the resolution set at 70,000, the AGC target at 1 × 10^6^ and the maximum injection time (max IT) at 120 ms. Metabolite identities were confirmed using two parameters: (1) the precursor ion *m/z* was matched within 5 ppm of theoretical mass predicted by the chemical formula; and (2) the retention time of metabolites was within 5% of the retention time of a purified standard run with the same chromatographic method. Chromatogram review and peak area integration were performed using Thermo Fisher software XCalibur Qual Browser, XCalibur Quan Browser software and Tracefinder 5.0. The peak area for each detected metabolite was normalized against the total ion count of that sample to correct any variations introduced from sample handling through instrument analysis.

Metabolite identification was performed using the Compound Discoverer software (v.3.2; Thermo Fisher). Metabolites were annotated at the MS2 level using both an inhouse mzVault spectral database curated from 1,051 authentic compound standards and the online spectral library mzCloud. The precursor mass tolerance was set to 5 ppm and fragment mass tolerance set to 10 ppm. Only metabolites with mzVault or mzCloud best match score above 50% and 75%, respectively, and retention time tolerance within 0.5 min to that of a purified standard run with the same chromatographic method, were exported to generate a list including compound names, molecular formula and retention time. The curated list was then used for further processing in the Tracefinder software (v.5.0; Thermo Fisher), in which extracted ion chromatographs for all compounds were examined and manually integrated if necessary. False positive, noise or chromatographically unresolved compounds were removed. The peak area for each detected metabolite was then normalized against the total ion count of that sample to correct any variations introduced from sample handling through instrument analysis. The normalized areas were used as variables for further statistical data analysis. Statistical analysis was performed using MetaboAnalyst (v.5.0)^60^. Metabolites were depicted using a heatmap based on *z*-scores of biological replicates.

### CoQ extraction and analysis

The CoQ redox state was determined by MS as previously described^22^.

### Seahorse XF mitochondrial stress test

BMDMs were plated at 200,000 cells per well in DMEM supplemented with 10% L929 supernatant in a 24-well Seahorse plate (Agilent) and treated with LPS for 3, 6 or 24 h. Compounds used in the assay were added to the appropriate ports of the injector plate at a final assay concentration of 5 μM oligomycin (port A), 2 μM FCCP (port B), 100 nM rotenone and 1 μM antimycin A (port C). The assay was performed on a Seahorse XF-24 flux analyser (Agilent) using the mito stress test programme^55^. Data were analysed using Seahorse Wave software (Agilent).

### ATP/ADP assay

ATP and ADP were measured using a luciferase-based assay that produces photons in proportion to ATP concentration as previously described^61,62^.

### Western blotting

Following treatment, cells were washed in PBS and lysed in 100 μl 1× Laemmli sample buffer (Bio-Rad) in RIPA buffer (Thermo Fisher) containing 4% β-mercaptoethanol and supplemented with 1 μl ml^−1^ benzonase nuclease (Sigma) and 1× cOmplete protease inhibitor (Sigma) cocktail. Protein was scraped and stored at −80 °C for future use.

Protein in 500 μl supernatant from 1 × 10^6^ cells was concentrated with 5 μl Strataclean Resin (Agilent) and vortexed for 1 min. The samples were then centrifuged at 210*g* for 2 min at 4 °C and the pellet was resuspended in RIPA buffer (Thermo Fisher) containing 1× Laemmli NuPAGE MES SDS running buffer, 4% β-mercaptoethanol and supplemented with 1 μl ml^−1^ benzonase nuclease (Sigma) and 1× cOmplete protease inhibitor (Sigma) cocktail.

All samples were resolved using 4–20% Bis-Tris Bolt gradient gels (Invitrogen), NuPAGE MES SDS running buffer (Thermo Fisher) and Precision Plus Protein pre-stained marker (Bio-Rad). Proteins were transferred onto a nitrocellulose membrane (Trans-Blot Turbo Transfer Pack (Invitrogen)) by semi-dry transfer using a Trans-Blot Turbo transfer system (Bio-Rad) at 2.5 A, 25 V for 7 min. Membranes were stained using Ponceau S and then washed with PBS-T (PBS with 0.05% Tween) or TBS-T (Tris-buffered saline (TBS) with 1% Tween). Membranes were then blocked in 5% (w/v) milk in PBS-T or 5% BSA in TBS-T for 1 h at room temperature. Membranes were incubated in primary antibody in 5% milk or 5% BSA in TBS-T for 5 min and then incubated in secondary antibodies in Intercept blocking buffer (Li-COR). Bands were imaged using Odyssey CLx instrument (Li-COR) or Amersham Imager 680 and quantified using Image Studio lite 2.5 (Li-COR).

### ASC cross-linking for western blotting

Following treatment, 3 × 10^6^ BMDMs per condition were washed in 200 μl cold 50 mM HEPES and then lysed in 200 µl lysis buffer (50 mM HEPES, 0.5% Triton X-100, 1 μl ml^−1^ benzonase nuclease, 1× cOmplete protease inhibitor cocktail and 1× phosSTOP cocktail (Sigma)). The samples were centrifuged at 6,000*g* for 15 min at 4 °C and 21 μl supernatant was collected and prepared for blotting by the addition of 7 μl 4× Laemmli NuPAGE MES SDS running buffer and 20 mM dithiothreitol. This sample was used to determine the total cellular ASC levels (ASC input). The pellet was washed by resuspension in 50 mM HEPES and centrifugation at 6,000*g* for 15 min at 4 °C. The pellet was then resuspended in 500 μl cross-linking buffer (50 mM HEPES and 150 mM NaCl). The cross-linker BS3 (bis(sulfosuccinimidyl)suberate) (Thermo Scientific) was added to each sample to a final concentration of 2 mM, the samples were inverted immediately and then incubated for 30 min at 45 °C. Following incubation, the samples were centrifuged at 6,000*g* for 15 min at 4 °C and the pellet were resuspended in 50 μl 1× Laemmli NuPAGE MES SDS running buffer with 20 mM dithiothreitol to assess the formation of ASC multimeric complex formation. All samples were boiled for 5 min at 95 °C before they were run on a 4–20% Bis-Tris Bolt gradient gels (Invitrogen).

### Real-time PCR

RNA was extracted using RNeasy Plus Mini kit (QIAGEN) according to the manufacturer’s instructions and quantified using a Nanodrop ND-8000 UV-visible spectrophotometer (Thermo Fisher). The isolated RNA was converted to complementary DNA (cDNA) using the High-Capacity cDNA Reverse Transcription kit (ThermoFisher Scientific).

Expression levels of *Tnf*, *Il10*, *Il1b* and *Rps18* were measured by real-time PCR using SYBR Green reagents and using primers designed inhouse and ordered from Merck, as detailed in Supplementary Table 1. Relative fold changes in expression were calculated using the comparative threshold cycle (C_t_) method and normalized to the housekeeping gene *Rps18*.

### Mitochondrial DNA copy number measurements

mtDNA copy number was quantified by digital-droplet PCR (ddPCR)^63^. Following LPS treatment, DNA was isolated from 2 × 10^6^ BMDMs using QIAGEN DNeasy Blood & Tissue Kit (69504) according to the manufacturer’s instructions. The DNA concentration of each sample was measured using a Nanodrop ND-8000 UV-visible spectrophotometer (Thermo Fisher) and adjusted to 10 ng μl^−1^, 1 ng μl^−1^ and 0.1 ng μl^−1^. A PCR mastermix with ddPCR Supermix for Probes (No dUTP) (1863023) containing primers and probes outlined in Supplementary Tables 2 and 3, respectively, was made up in a UV hood using pipettes, tips, plates and Eppendorf tubes that were sterilized under UV light for 30–45 min. A mixture of 1 μl DNA and a 21-μl reaction mixture containing primers (Supplementary Tables 2 and 3) and ddPCR Supermix for Probes (No dUTP) (1863023) was made up for each sample at 10 ng μl^−1^, 1 ng μl^−1^ and 0.1 ng μl^−1^ DNA and placed in a 96-well ddPCR plate (Bio-Rad). The plate was then sealed, vortexed for 20 s, spun down with a pulse spin and placed on a chilled block (4 °C) (Bio-Rad).

Droplets were generated using a Bio-Rad QX200 AutoDG droplet generator with corresponding Bio-Rad automated droplet-generating oil (1864110), Bio-Rad DG32 automated droplet generator cartridges (1864108) and Bio-Rad pipette tips for AutoDG system (1864120). Upon completion, the plate was removed and sealed with a foil seal (Bio-Rad; 1814040) using a plate sealer (Bio-Rad) set at 180 °C for 5 s. The plate was stored on ice until the next step.

ddPCR was run on a C1000 Touch Thermal Cycler (Bio-Rad) programmed to 95 °C for 30 s and 60 °C for 1 min repeated for 40 cycles followed by 90 °C for 10 min. Temperature change ramp rates were set at 2 °C s^−1^. The plate was stored at 4 °C until analysis.

Droplets were read by a QX200 Droplet Reader (Bio-Rad) using the following experimental settings: absolute quantification, rare event detection, copy number variation and supermix for probes with no dUTP. The results were analysed using QuantaSoft analysis software (Bio-Rad) and the average mtDNA copy number (HEX probe) was normalized to the nuclear DNA copy number (FAM probe).

### Enzyme-linked immunosorbent assay

The concentration of cytokines in extracellular medium was measured using ELISA Duoset kits for mouse IL-10 (DY417), TNF (DY410), IL-6 (DY406) and IL-1β (DY401) according to the manufacturer’s instructions. Absorbance was measured at 450 nm using a microplate reader (SpectraMax Plus 384 Microplate Reader). Concentrations were calculated using the corresponding standard curve after accounting for the twofold dilution of sample in the assay.

### Nitric oxide production measurements

Nitric oxide production was assessed by measuring extracellular nitrite levels using the Promega Griess assay (G2930) according to the manufacturer’s instructions.

### LDH assay

LDH release from cells was measured using the CytoTox 96 Non-Radioactive Cytotoxicity Assay (Promega) according to the manufacturer’s instructions using 50 μl extracellular medium. Cell medium was used as a blank to correct for background absorbance and all measurements were normalized to LDH release from nonstimulated BMDMs.

### Live cell confocal microscopy

BMDMs were plated in an eight-well chambered coverslip (ibidi) at 180,000 cells per well and treated according to the experimental setup. Images were acquired using a Zyla 4.2 PLUS sCMOS camera attached to an Andor DragonFly 500 confocal spinning disk mounted on a Nikon Eclipse TiE microscope using a CFI Plan Apochormat lambda ×100 oil immersion objective. Images were acquired at physiological conditions (37 °C, 5% CO_2_ and 95% relative humidity) within a H301-K-FRAME stage top incubator (Okolab), using the Fusion user interface (Andor) and analysed using Fiji ImageJ. The image processing workflow was automated using the following Fiji ImageJ macro scripts:

1. batch_FlatR: uses onboard tools in Fiji to batch process Z-stack multichannel image data and returns maximum intensity projection multichannel images as tiff files.
2. batchsplitR: uses maximum intensity projection multichannel images in tiff format as input and returns single-channel images as separate tiff files for further processing.
3. modifyR: uses onboard tools in Fiji to enhance contrast by histogram equalization with the same values across a batch of images. The macro further uses the Smooth function to reduce noise across the same batch of images. This macro is used specifically to limit any cosmetic alterations to individual images that might skew the final intensity measurement.
4. measureR_Indexed: this macro limits the measurement parameters in Fiji to calculate the intensity values using pixel values across a batch of preselected ROIs. The macro returns an excel sheet tabulating the mean, minimum, maximum, integrated density and raw integrated density for all the pixel values within the batch of ROIs.

### Mitochondrial reactive oxygen species assessment

Following treatment, BMDMs were incubated in 2.5 μM MitoSOX Red, 5 μM MitoNeoD or 5 μM NeoD and 5 nM MitoTracker Deep Red FM in DMEM (10% FBS and 1% pen–strep) for 20 min. Alternatively, BMDMs were incubated with 5 μM hydroethidine, 1 μM Hoechst and 5 nM MitoTracker Deep Red FM in DMEM (10% FBS and 1% pen–strep) for 20 min. The cells were then washed with PBS and the medium was replaced with phenol red-free DMEM (Gibco). All images were acquired with the Andor spinning disk confocal described above, using a 500-ms exposure, 50% laser intensity and excitation/emission 561/620 nm for ROS probes, 500 ms exposure, 50% laser intensity and excitation/emission 637/700 nm for MitoTracker Deep Red FM and 100-ms exposure, 20% intensity and excitation/emission 405/450 nm for Hoechst. Ten Z-stacks of 0.2 μm were acquired. The absolute fluorescent intensity of 50–80 cells per biological replicate was quantified with fluorescence intensity measured as absolute intensity with arbitrary units (a.u.).

### Mitochondrial membrane potential assessment

Following treatment, BMDMs were incubated in 10 nM TMRM and 5 nM MitoTracker Deep Red FM in DMEM (10% FBS and 1% pen–strep) for 20 min and the medium was then replaced with phenol red-free DMEM (Gibco). All images were acquired with the Andor spinning disk confocal described above, using 500-ms exposure, 50% laser intensity and excitation/emission 561/620 nm for TMRM and 500-ms exposure, 50% laser intensity and excitation/emission 637/700 nm for MitoTracker Deep Red FM. Ten Z-stacks of 0.2 μm were acquired. The absolute intensity of TMRM fluorescence was measured by selecting 20 ROIs of 15 μm × 15 μm per biological replicate. This was normalized to the corresponding absolute intensity (a.u.) of MitoTracker Deep Red ROI to quantify ∆ψ_m_.

### Plasma membrane potential assessment

FLIPR membrane potential assay kit (Molecular Devices, catalogue number R-8042), referred to as plasma membrane potential indicator (PMPI) was used to measure ∆ψ_p_^62^. Component A was resuspended in 10 ml water to make a 100× stock solution. Cells were incubated in 5 nM MitoTracker Deep Red FM in DMEM (10% FBS and 1% pen–strep) for 10 min, which was then removed and replaced with 350 μl 1× PMPI immediately before image acquisition. To depolarize the plasma membrane KCl was added to the cells at a final concentration of 25 mM and images were immediately acquired. All images were acquired with the Andor spinning disk confocal described above, using 100-ms exposure, 10% laser intensity and excitation/emission 488/525 nm for PMPI and 500-ms exposure, 50% laser intensity and excitation/emission 637/700 nm for MitoTracker Deep Red FM. Ten Z-stacks of 0.2 μm were acquired. The absolute fluorescent intensity (a.u.) of 15–20 cells per biological replicate was quantified.

### Immunofluorescence

Immunofluorescence was performed as described previously^64^. BMDMs were incubated in primary antibodies at 1:500 in 5% FBS for 2 h at room temperature for TOMM20 (11802-1-AP) or overnight at 4 °C for ATP synthase (MAB3494) and TOMM20 (ab232589). The cells were mounted onto glass slides using mounting medium (Dako for spinning disk confocal microscopy or ProLong Diamond for super-resolution microscopy) and left to dry for 12 h at room temperature and then stored at 4 °C until imaging.

### Super-resolution microscopy

Fixed and live cell super-resolution images were acquired with a Nikon SIM microscope using a SR Apo TIRF ×100 1.49 NA oil objective and a DU897 Ixon camera (Andor). Seven Z-stacks of 0.2 μm each were acquired. The images were computationally reconstructed with the standard modal reconstruction settings for Z-stack on the NIS‐Elements software (Nikon). For live-cell imaging, the cells were incubated in 5 nm MitoTracker Green for 20 min, washed and imaged under physiological conditions (37 °C, 5% CO_2_ and 95% humidity) for the duration of imaging. Nine Z-stacks of 0.2 μm each were acquired. The images were computationally reconstructed with modified reconstruction settings for live samples to increase illumination modulation contrast and reduce out of focus blur on the NIS‐Elements software (Nikon). The images were further processed for clarity by background subtraction (rolling-ball 50) and 1× smoothening on FIJI.

Fixed cell super-resolution images for analysis of mitochondrial morphology in 3D were obtained with the Zeiss Elyra7 lattice (SIM), using the Plan-Apochromat ×63/1.4 Oil DIC M27 objective with 15 phases and 0.091-μm intervals. Images were acquired with 20-ms exposure time with 405 nm (20.0%), 488 nm (4.0%) and 561 nm (6.0%) lasers. Standard deconvolution was performed in Zen Black.

### Quantification of morphological features

Mitochondrial morphology was assessed in the ROIs (15 × 15 μm) of BMDMs stained with MitoTracker Deep Red from images obtained by live-cell confocal microscopy. Morphological features were analysed and quantified using an automated processing workflow called MitoMAPR^65^. In this process, ROIs were converted to binary images, which were skeletonized and processed with the AnalyzeSkeleton plugin.

For analysis of mitochondrial morphology in three-dimensions (3D) and quantification of ATP synthase puncta from SIM images obtained as described, individual cells were cropped in ImageJ (Fiji, National Institutes of Health). Using Imaris v.10.1.0 (Oxford Instruments), objects in separate channels were segmented and rendered in 3D with the surfaces function. Segmentation setup included smoothing with surfaces detail of 0.0986 µm and background subtraction (local contrast) of 0.370 and 0.2 µm were used for TOMM20 and ATP synthase channels, respectively. Machine-learning segmentation was used for Hoechst channel with smoothing and surfaces detail of 1 µm. Manual thresholding was used for TOMM20 and ATP synthase channels. For ATP synthase channel, split touching objects (region growing) was enabled with an intensity-based seed points diameter of 0.2 µm. A surfaces filter was then applied to select objects above ten voxels. Object statistics from Imaris surfaces were then recorded in Microsoft Excel.

### LPS-induced inflammation model

Female and male mice (11–12 weeks old) littermates were randomly assigned to experimental groups. Mice were injected intraperitoneally with PBS or LPS from *E.* *coli* O55:B5 (2.5 mg kg^−1^, Sigma) at a volume of 100 μl per injection. After 2 h, mice were killed in a CO_2_ chamber and blood was collected from the vena cava. Blood was centrifuged at 10,000*g* for 10 min at 4 °C and serum cytokines were analysed using a V-PLEX pro-inflammatory panel mouse kit (Meso Scale Discovery).

### MSU-induced peritonitis

Female mice (7–13 weeks old) were randomly assigned to experimental groups. Mice were injected intraperitoneally with PBS or MSU crystals (30 mg kg^−1^, suspended in PBS, Invivogen) for 6 h. Mice were then killed in a CO_2_ chamber and a peritoneal lavage was performed using 2.5 ml PBS. The lavage fluid was collected and cells were pelleted by centrifugation at 350*g* for 5 min at 4 °C and resuspended to a concentration of 1 × 10^6^ cells per 100 μl in eBioscience Fixable Viability Dye eFluor 450 (1:1,000, Thermo Fisher) for 15 min on ice. Cells were then washed in PBS by centrifugation at 350*g* for 5 min at 4 °C followed by incubation in anti-CD16/CD32 (1 μg in 50 μl 1% FBS (PBS); 101302, BioLegend) for 10 min on ice. The following antibodies were then added at 0.25 μg each in 50 μl 1% FBS (PBS): APC anti-mouse CD45 (103111, BioLegend), FITC anti-mouse CD11b (101205, BioLegend), PE anti-mouse Ly6G (127607, BioLegend). Cells were incubated for a further 30 min in the dark on ice, washed twice with 1% FBS (PBS) and then resuspended in 500 μl 1% FBS (PBS) for analysis using a BD LSRFortessa Cell Analyser. Live cells were identified as eBioscience Fixable Viability Dye eFluor 450 positive and neutrophils as CD45^+^CD11b^+^Ly6G^+^ cells. Data were analysed using FlowJo software v.10.10.0 (FlowJo).

### Statistical analysis

All data are presented as mean ± s.e.m. of biological replicates. Statistical analysis was performed using Prism 9.0 (GraphPad). *P* values were calculated using a two-tailed Student’s *t*-test for paired comparisons or one-way or two-way analysis of variance (ANOVA) for multiple comparisons. Multiple comparisons were corrected for using the Dunnett method for one-way ANOVA, Tukey method for two-way ANOVA and Holm–Šidák method for multiple *t*-tests.

### Reporting summary

Further information on research design is available in the Nature Portfolio Reporting Summary linked to this article.

## Supplementary information

Supplementary Information Supplementary Tables 1–3.

Reporting Summary

Supplementary Video 1 Representative 3D Imaris rendering of nonstimulated BMDMs. Mitochondria labelled with TOMM20 (green) and ATP synthase (magenta) antibodies and nucleus stained with Hoechst (blue). Scale bar, 5 µm.

Supplementary Video 2 Representative 3D Imaris rendering of BMDMs stimulated with LPS for 24 h. Mitochondria labelled with TOMM20 (green) and ATP synthase (magenta) antibodies and nucleus stained with Hoechst (blue). Scale bar, 5 µm.

## Source data

Source Data Fig. 1 Statistical Source Data.

Source Data Fig. 2 Statistical Source Data.

Source Data Fig. 3 Statistical Source Data.

Source Data Fig. 4 Statistical Source Data.

Source Data Fig. 4 Unprocessed western blots.

Source Data Extended Data Fig. 1 Statistical Source Data.

Source Data Extended Data Fig. 2 Statistical Source Data.

Source Data Extended Data Fig. 2 Unprocessed western blots.

Source Data Extended Data Fig. 3 Statistical Source Data.

Source Data Extended Data Fig. 3 Unprocessed western blots.

Source Data Extended Data Fig. 4 Statistical Source Data.

Source Data Extended Data Fig. 4 Unprocessed western blots.

Source Data Extended Data Fig. 5 Statistical Source Data.

Source Data Extended Data Fig. 6 Statistical Source Data.

Source Data Extended Data Fig. 7 Statistical Source Data.

Source Data Extended Data Fig. 8 Statistical Source Data.

Source Data Extended Data Fig. 8 Unprocessed western blots.

Source Data Extended Data Fig. 9 Statistical Source Data.

Source Data Extended Data Fig. 10 Statistical Source Data.

Source Data Extended Data Fig. 10 Unprocessed western blots.

## Data Availability

Source data are provided with this paper.
