# Supplementary material for: Pro-inflammatory macrophages produce mitochondria-derived superoxide by reverse electron transport at complex I that regulates IL-1β release during NLRP3 inflammasome activation
Source: Nat Metab. 2025 Feb 19;7(3):493–507. doi: 10.1038/s42255-025-01224-x (PMC11946910; doi:10.1038/s42255-025-01224-x)
Supplement: Supplementary file 1 — Supplementary Tables 1–3. [file 42255_2025_1224_MOESM1_ESM.pdf]

nature metabolism

Supplementary information https://doi.org/10.1038/s42255-025-01224-x

Pro-inflammatory macrophages produce mitochondria-derived superoxide by reverse electron transport at complex I that regulates IL-1β release during NLRP3 inflammasome activation

In the format provided by the authors and unedited

Supplementary Table 1: qPCR primers

Primer	Forward (5'→ 3')	Reverse (5'→ 3')
IL1B	TGCCACCTTTTGACAGTGATG	TGATGTGCTGCTGCGAGATT
TNF	GATCGGTCCCCAAAGGGATG	TGAGAAGATGATCTGAGTGTGAG
IL10	GGCGCTGTCATCGATTTCTC	ATGGCCTTGTAGACACCTTGG
RPS18	TGGGAACTTCTCATCCCTTTG	GGATGTGAAGGATGGGAAGT

Supplementary Table 2: ddPCR primer sequences

**Gene**	**Sequence**
ND1 forward	GAGCCTCAAACTCCAAATACTCACT
ND1 reverse	GAACTGATAAAAGGATAATAGCTATGGTTACTTCA
actin forward	CTGCTCTTTCCCAGACGAGG
actin reverse	AAGGCCACTTATCACCAGCC

Supplementary Table 3: ddPCR probe sequences

**Gene/Probe/Quencher**	**Sequence**
β2m/FAM/BHQ1	5'6-FAM/ATTGCCTTTCTGACTAGGTG/3BHQ_1
ND1/HEX/BHQ1	5'HEX/CCGTAGCCCAAACAAT/3BHQ_1
