# Supplementary material for: Pro-inflammatory macrophages produce mitochondria-derived superoxide by reverse electron transport at complex I that regulates IL-1β release during NLRP3 inflammasome activation
Source: Nat Metab. 2025 Feb 19;7(3):493–507. doi: 10.1038/s42255-025-01224-x (PMC11946910; doi:10.1038/s42255-025-01224-x)
Supplement: Supplementary file 2 — Reporting Summary [file 42255_2025_1224_MOESM2_ESM.pdf]

nature portfolio

Corresponding author(s): Michael P. Murphy

Last updated by author(s): 17/12/2024

Reporting Summary

Nature Portfolio wishes to improve the reproducibility of the work that we publish. This form provides structure for consistency and transparency in reporting. For further information on Nature Portfolio policies, see our Editorial Policies and the Editorial Policy Checklist.

Statistics

For all statistical analyses, confirm that the following items are present in the figure legend, table legend, main text, or Methods section.

n/a	Confirmed	
☐	☒	The exact sample size (*n*) for each experimental group/condition, given as a discrete number and unit of measurement
☐	☒	A statement on whether measurements were taken from distinct samples or whether the same sample was measured repeatedly
☐	☒	The statistical test(s) used AND whether they are one- or two-sided<br>*Only common tests should be described solely by name; describe more complex techniques in the Methods section.*
☒	☐	A description of all covariates tested
☐	☒	A description of any assumptions or corrections, such as tests of normality and adjustment for multiple comparisons
☐	☒	A full description of the statistical parameters including central tendency (e.g. means) or other basic estimates (e.g. regression coefficient) AND variation (e.g. standard deviation) or associated estimates of uncertainty (e.g. confidence intervals)
☐	☒	For null hypothesis testing, the test statistic (e.g. *F*, *t*, *r*) with confidence intervals, effect sizes, degrees of freedom and *P* value noted<br>*Give P values as exact values whenever suitable.*
☒	☐	For Bayesian analysis, information on the choice of priors and Markov chain Monte Carlo settings
☒	☐	For hierarchical and complex designs, identification of the appropriate level for tests and full reporting of outcomes
☒	☐	Estimates of effect sizes (e.g. Cohen's *d*, Pearson's *r*), indicating how they were calculated

Our web collection on statistics for biologists contains articles on many of the points above.

Software and code

Policy information about availability of computer code

Data collection	Peak area integration for metabolomics was performed using LabSolutions software (Shimadzu, UK) or using the ThermoFisher software XCalibur Qual Browser, XCalibur Quan Browser software and Tracefinder 5.0. Metabolite identification was performed using the Compound Discoverer software (v.3.2; ThermoFisher). The peak area for UQ9 and UQ9H2 was quantified using MassLynx 4.1 software.
Data analysis	Mass spectrometry statistical analysis was performed using MetaboAnalyst (v 5.0). Mitochondrial stress test data was analysed using Seahorse Wave software (Agilent). Western blot bands were quantified using Image Studio lite 2.5 (Li-COR). ddPCR results were analysed using QuantaSoft analysis software (BioRad). Super resolution images were computationally reconstructed using the NIS-Elements software (Nikon). For fixed cell super resolution images standard deconvolution was performed in Zen Black. Confocal microscopy images were analysed using Fiji ImageJ2 (version 2..3.0). Flow cytometry data analysed using FlowJo software version 10.10.0 (FlowJo LLC). Statistical analysis was performed using Prism 9.0 (Graphpad).

For manuscripts utilizing custom algorithms or software that are central to the research but not yet described in published literature, software must be made available to editors and reviewers. We strongly encourage code deposition in a community repository (e.g. GitHub). See the Nature Portfolio guidelines for submitting code & software for further information.

Data

Policy information about availability of data

All manuscripts must include a data availability statement. This statement should provide the following information, where applicable:

- Accession codes, unique identifiers, or web links for publicly available datasets
- A description of any restrictions on data availability
- For clinical datasets or third party data, please ensure that the statement adheres to our policy

Source data are provided with this paper.

Research involving human participants, their data, or biological material

Policy information about studies with human participants or human data. See also policy information about sex, gender (identity/presentation), and sexual orientation and race, ethnicity and racism.

Reporting on sex and gender	N/A
Reporting on race, ethnicity, or other socially relevant groupings	N/A
Population characteristics	N/A
Recruitment	N/A
Ethics oversight	N/A

Note that full information on the approval of the study protocol must also be provided in the manuscript.

Field-specific reporting

Please select the one below that is the best fit for your research. If you are not sure, read the appropriate sections before making your selection.

☒ Life sciences ☐ Behavioural & social sciences ☐ Ecological, evolutionary & environmental sciences

For a reference copy of the document with all sections, see nature.com/documents/nr-reporting-summary-flat.pdf

Life sciences study design

All studies must disclose on these points even when the disclosure is negative.

Sample size	No statistical methods were used to determine sample sizes. However, a minimum of 3 biological replicates was used per experiment to account for biological variability. The 3 Rs principle was taken into account when using primary murine bone-marrow derived macrophages and in vivo experiments
Data exclusions	Data were not excluded from the study.
Replication	The number of biological replicates is indicated in the figure legends, where each data point represents one individual mouse or a biological replicate of treated cells
Randomization	All samples, including for in vivo experiments were randomly divided into groups for treatment.
Blinding	Blinding was not used for either in vitro or in vivo experiments for practical reasons and due to a limited number of investigators.

Reporting for specific materials, systems and methods

We require information from authors about some types of materials, experimental systems and methods used in many studies. Here, indicate whether each material, system or method listed is relevant to your study. If you are not sure if a list item applies to your research, read the appropriate section before selecting a response.

Materials & experimental systems

n/a	Involved in the study
☐	☒ Antibodies
☐	☒ Eukaryotic cell lines
☒	☐ Palaeontology and archaeology
☐	☒ Animals and other organisms
☒	☐ Clinical data
☒	☐ Dual use research of concern
☒	☐ Plants

Methods

n/a	Involved in the study
☒	☐ ChIP-seq
☐	☒ Flow cytometry
☒	☐ MRI-based neuroimaging

Antibodies

Antibodies used

Working dilutions of antibodies were 1:1,000 unless otherwise stated. Anti-ASC (67824), ATPIF1 (8528), Caspase-1 (E2Z1C) (24232), Cleaved Caspase-1 (Asp296) (89332), Cleaved gasdermin D (Asp276) (10137), Cleaved IL-1β (Asp117) (63124), IL-1β (12507), GAPDH (5174), Gasdermin D (E9S1X) (39754), Hexokinase I (2024T), NLRP3 (15101), PKM1/2 (3910), PKM2 (4053T), pyruvate dehydrogenase (3205) and VDAC (4661) antibodies were purchased from Cell Signalling. Anti-IRG1 (ab222411), ATP Synthase (MAB3494; 1:500) and TOMM20 (ab232589; 1:500) antibodies and OXPHOS blue native antibody cocktail (1:500; ab110412) were purchased from Abcam. Anti-Tubulin (T9026) and vinculin (SAB4200729) antibodies were purchased from Sigma-Aldrich. Anti-TOMM20 (11802-1-AP) was purchased from Proteintech. Mouse IgG (926-68070) and rabbit IgG (926-3211) antibodies both used at 1:15,000 were purchased from Li-cor and used for detection of all primary antibodies except cleaved gasdermin D. Cleaved gasdermin D was detected using anti-rabbit IgG (W4011) purchased from Promega and analysed using Amersham ECL prime western blotting detection reagents (RPN2232; Cytiva).
Fluorescent antibodies A-21125 (Invitrogen), 4412S (Cell signalling) and Alexa Fluor™ 568 rabbit IgG (A-11036; ThermoFisher) were all used at 1:1,000.

Validation

All antibodies were used according to manufacturer's instructions. Antibody validation on manufacturer's website is as follows:
Anti-ASC (67824): by western blot analysis of extracts from J774A.1 and Raw 264.7 cells. The expected band appeared at the expected molecular weight.
ATPIF1 (8528): by western blot analysis of extracts from HeLa, DLD-1, K-562, ZR-75-1 cells. The expected band appeared at the expected molecular weight.
Caspase-1 (E2Z1C) (24232): by western blot analysis of extracts from murine bone-marrow derived macrophages and EL4 cell line. The expected band appeared at the expected molecular weight.
Cleaved Caspase-1 (Asp296) (89332): by western blot analysis of extracts from murine bone-marrow derived macrophages and EL4 and M1 cell lines. The expected band appeared at the expected molecular weight.
Cleaved gasdermin D (Asp276) (10137): by western blot analysis of extracts from murine bone-marrow derived macrophages. The expected band appeared at the expected molecular weight.
Cleaved IL-1β (Asp117) (63124): by western blot analysis of extracts from murine bone-marrow derived macrophages. The expected band appeared at the expected molecular weight.
IL-1β (12507: by western blot analysis of extracts from RAW264.7 cells. The expected band appeared at the expected molecular weight.
GAPDH (5174: by western blot analysis of extracts from HeLa, NIH3T3, C6 and COS-7 cell lines. The expected band appeared at the expected molecular weight.
Gasdermin D (E9S1X) (39754): by western blot analysis of extracts from murine bone-marrow derived macrophages and gasdermin D knockout PC-3 cells.
Hexokinase I (2024T): by western blot analysis of extracts from several cell lines. The expected band appeared at the expected molecular weight.
NLRP3 (15101): by western blot analysis of extracts from bone marrow derived dendritic cells, THP-1 cells and other cell lines. The expected band appeared at the expected molecular weight.
PKM1/2 (3190): by western blot analysis of extracts from several cell lines. The expected band appeared at the expected molecular weight.
PKM2 (4053T): by western blot analysis of extracts from several cell lines and mouse skeletal muscle. The expected band appeared at the expected molecular weight.
pyruvate dehydrogenase (3205): by western blot analysis of extracts from several cell lines. The expected band appeared at the expected molecular weight.
VDAC (4661): by western blot analysis of extracts from several cell lines. The expected band appeared at the expected molecular weight.
Anti-IRG1 (ab222411): by western blot analysis of extracts from THP-1 and RAW 264.7 cells. The expected band appeared at the expected molecular weight.
ATP Synthase (MAB3494): by immunocytochemistry and immunoprecipitation in rat neurons and glia.
TOMM20 (ab232589): by immunofluorescence and immunocytochemistry in HeLa cells and mouse small intestine.
OXPHOS blue native antibody cocktail (ab110412): in complex I knockout fibroblasts and in mouse primary neuronal cells.
Anti-Tubulin (T9026): by western blot analysis of extracts from several cell lines. The expected band appeared at the expected molecular weight.
Vinculin (SAB4200729): by western blot analysis of extracts from several cell lines. The expected band appeared at the expected molecular weight.
Anti-TOMM20 (11802-1-AP): by immunofluorescence in HUVEC, HepG2 and C6 cells.

Eukaryotic cell lines

Policy information about cell lines and Sex and Gender in Research

Cell line source(s)	THP-1 cell lines were purchased from American Type Culture Collection (ATCC). Primary bone marrow derived cells were acquired from male mice.
Authentication	The cell lines have been characterized previously PMID: 33380493
Mycoplasma contamination	Cell lines tested negative for mycoplasma contamination prior to use.
Commonly misidentified lines (See ICLAC register)	N/A

Animals and other research organisms

Policy information about studies involving animals; ARRIVE guidelines recommended for reporting animal research, and Sex and Gender in Research

Laboratory animals	Wildtype (WT) mice (C57BL/6J) were purchased from Charles River Laboratories, UK. The ND6P25L mouse strain59 was generously provided by Professor Douglas Wallace, University of Pennsylvania and backcrossed onto the C57BL/6J background. C57Bl/6J mice carrying a single copy of Ciona intestinalis AOX gene in the Rosa26 locus were generated by T. Braun, H. T. Jacobs and M. Szibor23. Mice were bred and maintained in pathogen-free facilities with a 12 h:12 h light:dark cycle, a room temperature of 19 °C to 22 °C, relative humidity 55% ± 10%, and with ad lib food and water.
Wild animals	This study did not involve wild animals.
Reporting on sex	Bone marrow used to generate bone marrow derived macrophages was acquired from male mice only. Both male and female mice were used for in vivo mouse experiments.
Field-collected samples	This study did not involve field-collected samples.
Ethics oversight	All mouse experiments were carried out in accordance with the UK Animals (Scientific Procedures) Act, 1986 (Home Office PPL no. P6C97520A and PP1730969). All procedures were approved by the University of Cambridge Animal Welfare and Ethical Review Body (AWERB) Committee.

Note that full information on the approval of the study protocol must also be provided in the manuscript.

Plants

Seed stocks	N/A
Novel plant genotypes	N/A
Authentication	N/A

Flow Cytometry

Plots

Confirm that:

- [x] The axis labels state the marker and fluorochrome used (e.g. CD4-FITC).
- [x] The axis scales are clearly visible. Include numbers along axes only for bottom left plot of group (a 'group' is an analysis of identical markers).
- [x] All plots are contour plots with outliers or pseudocolor plots.
- [x] A numerical value for number of cells or percentage (with statistics) is provided.

Methodology

Sample preparation	The lavage fluid was collected and cells were pelleted by centrifugation at 350 × g for 5 min at 4°C and resuspended to a concentration of 1 x 106 cells per 100 µL in eBioscience™ Fixable Viability Dye eFluor™ 450 (1/1000, ThermoFisher) for 15 min on ice. Cells were then washed in PBS by centrifugation at 350 × g for 5 min at 4 °C followed by incubation in anti-CD16/

	CD32 (1 µg in 50 µL 1% FBS (PBS); 101302, Biolegend) for 10 min on ice. The following antibodies were then added at 0.25 µg each in 50 µL 1% FBS (PBS): APC anti-mouse CD45 (103111, Biolegend), FITC anti-mouse CD11b (101205, Biolegend), PE anti-mouse Ly6G (127607, Biolegend). Cells were incubated for a further 30 min in the dark on ice, washed twice with 1% FBS (PBS) and then resuspended in 500 µL 1% FBS (PBS) for analysis.
Instrument	BD LSRFortessa™ Cell Analyzer
Software	Data was analysed using FlowJo software version 10.10.0 (FlowJo LLC).
Cell population abundance	No sorting was done.
Gating strategy	Gating strategy is shown in extended data figure 10o

☒ Tick this box to confirm that a figure exemplifying the gating strategy is provided in the Supplementary Information.
