# Supplementary figures and images for: Pro-inflammatory macrophages produce mitochondria-derived superoxide by reverse electron transport at complex I that regulates IL-1β release during NLRP3 inflammasome activation
Source: Nat Metab. 2025 Feb 19;7(3):493–507. doi: 10.1038/s42255-025-01224-x (PMC11946910; doi:10.1038/s42255-025-01224-x)

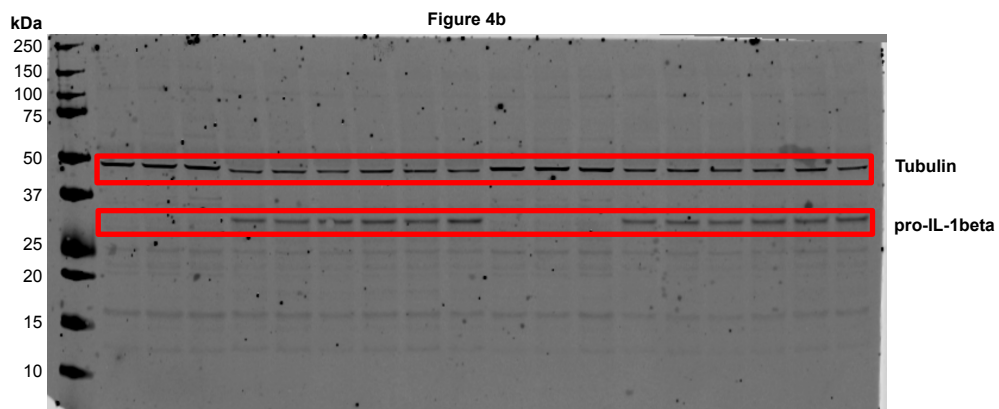

**Figure 4b - anti-pro-IL-1beta**

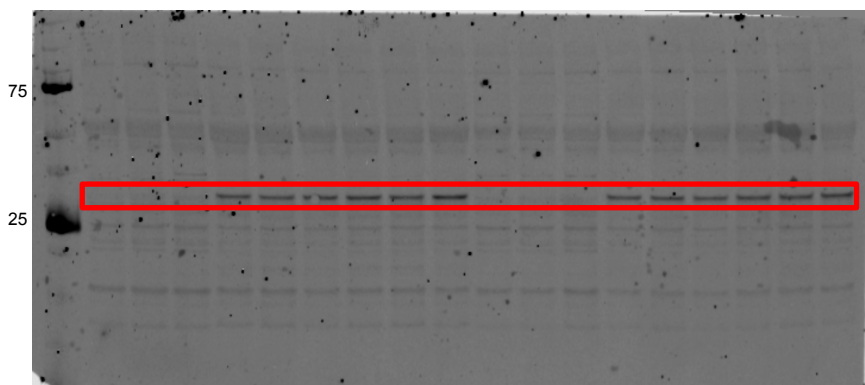

**Figure 4b - anti-tubulin**

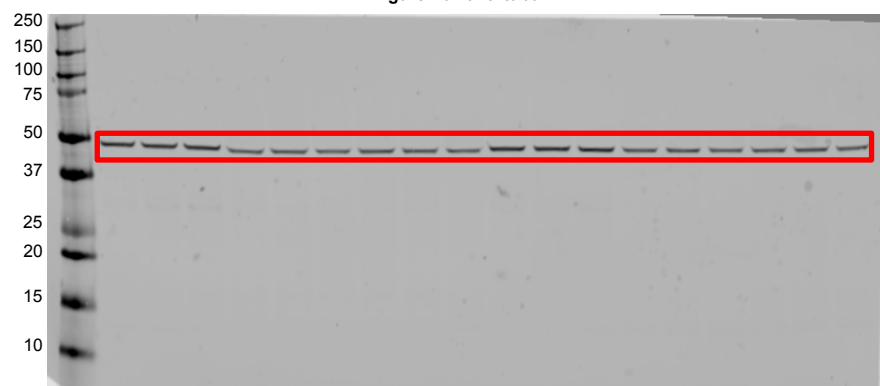

Supplement: Supplementary file 9 — Unprocessed western blots. [file 42255_2025_1224_MOESM9_ESM.pdf]

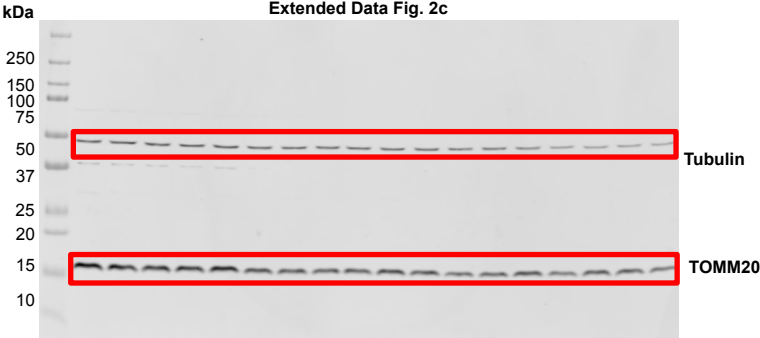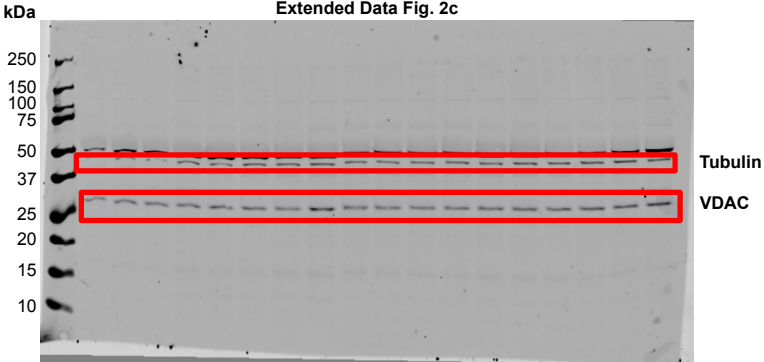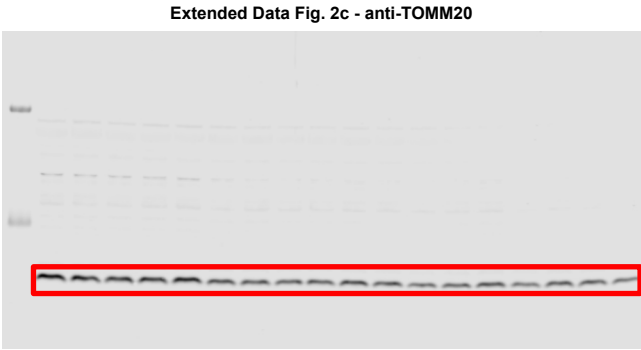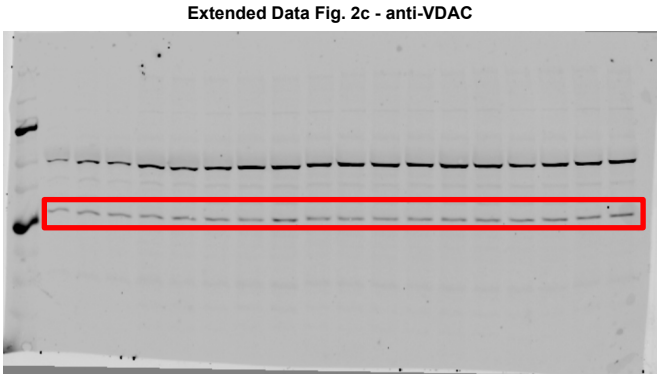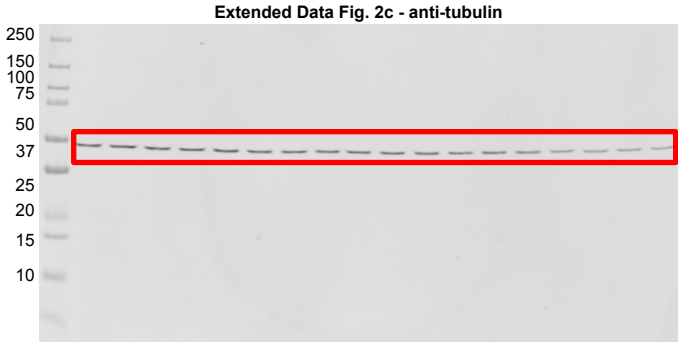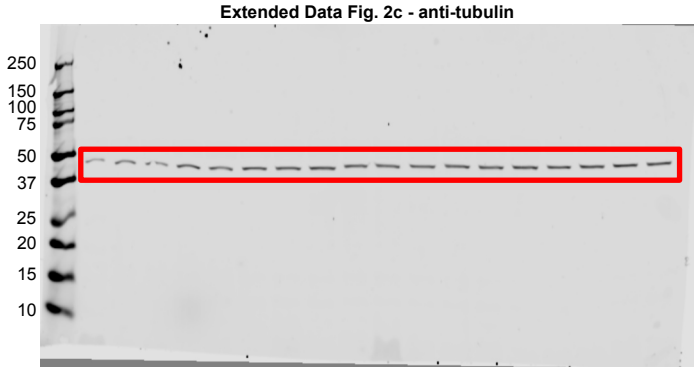

Supplement: Supplementary file 12 — Unprocessed western blots. [file 42255_2025_1224_MOESM12_ESM.pdf]

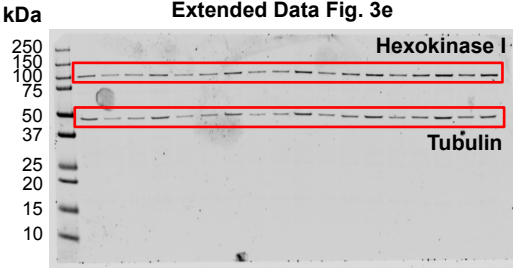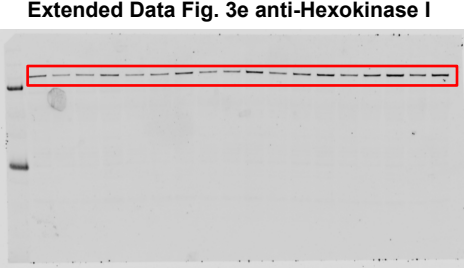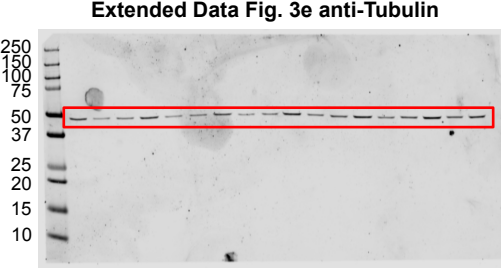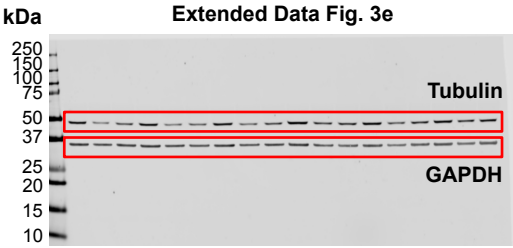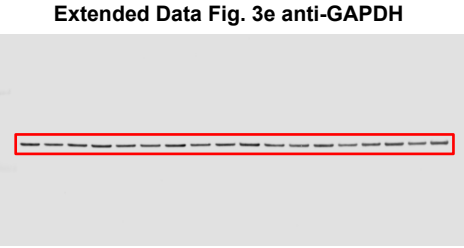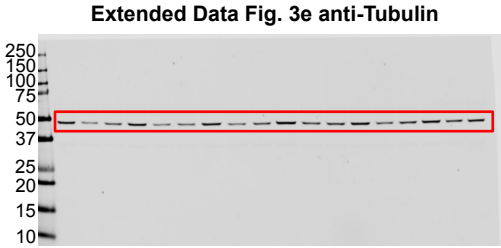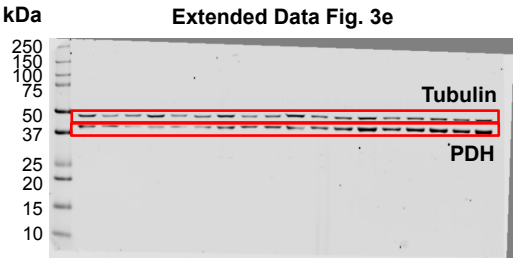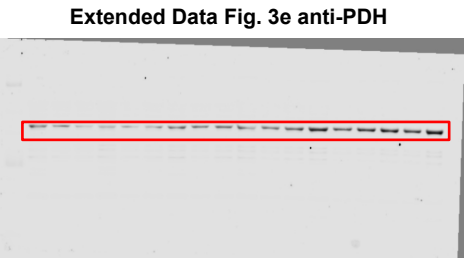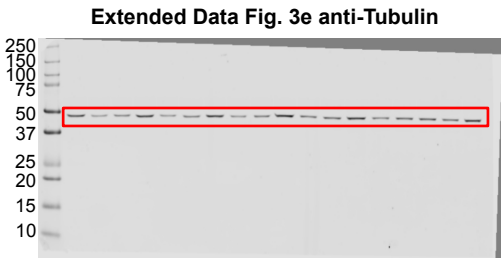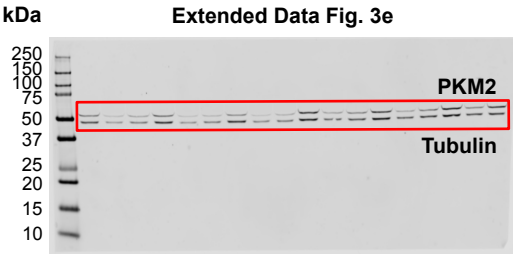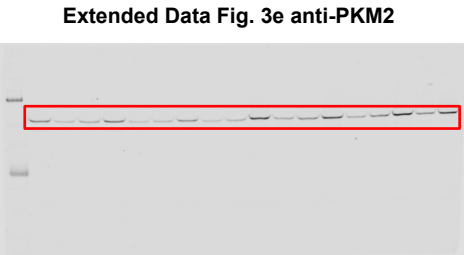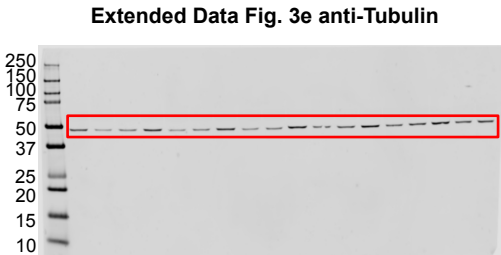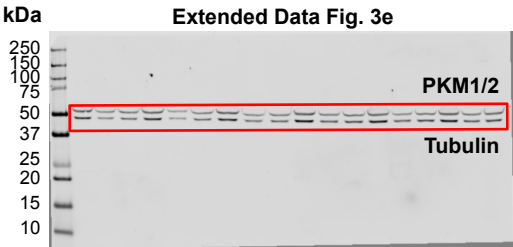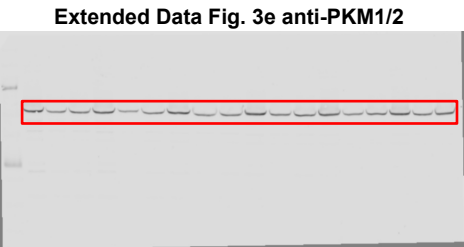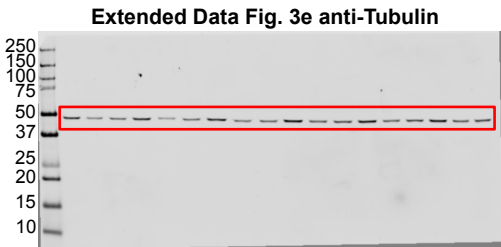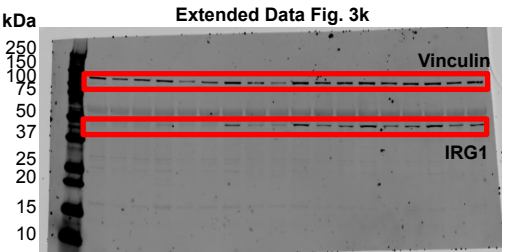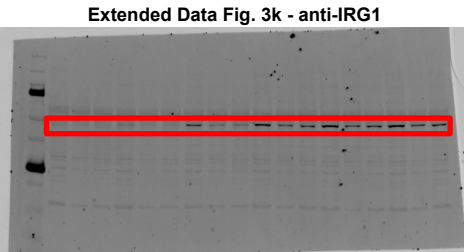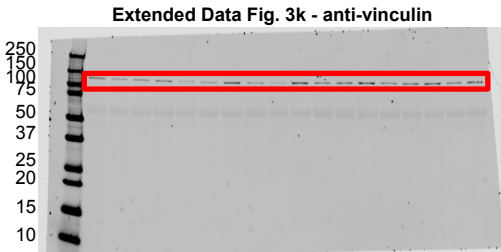

Supplement: Supplementary file 14 — Unprocessed western blots. [file 42255_2025_1224_MOESM14_ESM.pdf]

Extended Data Fig. 8c

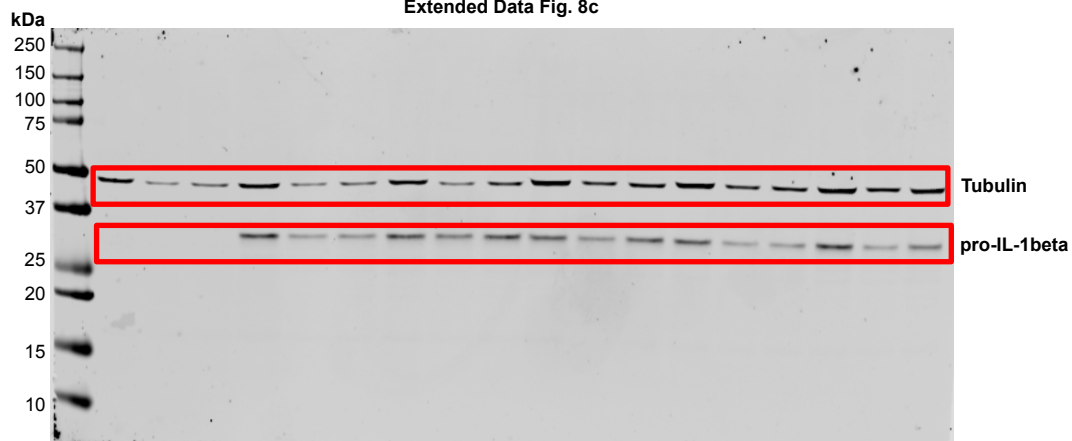

Extended Data Fig. 8c - anti-pro-IL-1beta

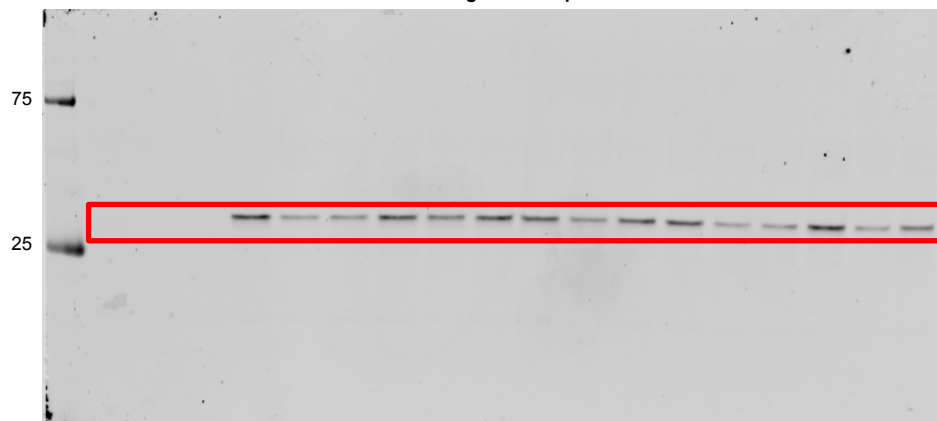

Extended Data Fig. 8c - anti-tubulin

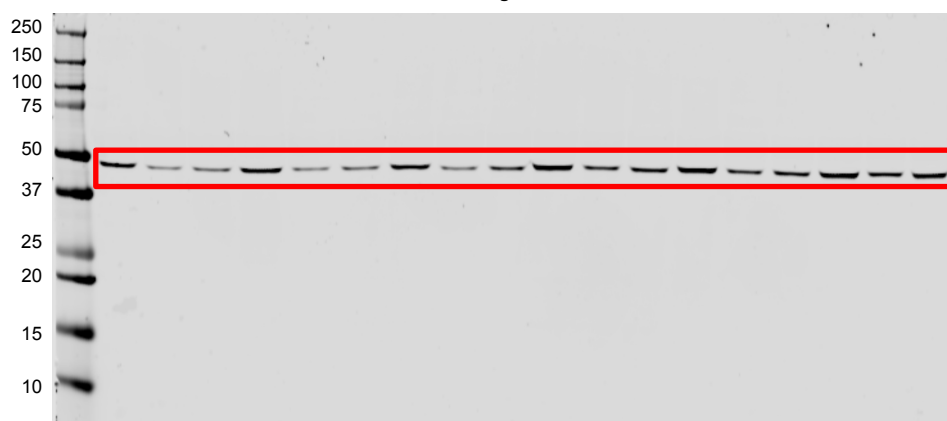

Supplement: Supplementary file 21 — Unprocessed western blots. [file 42255_2025_1224_MOESM21_ESM.pdf]
