# Supplementary material for: Pro-inflammatory macrophages produce mitochondria-derived superoxide by reverse electron transport at complex I that regulates IL-1β release during NLRP3 inflammasome activation
Source: Nat Metab. 2025 Feb 19;7(3):493–507. doi: 10.1038/s42255-025-01224-x (PMC11946910; doi:10.1038/s42255-025-01224-x)

Extended Data Fig. 4a

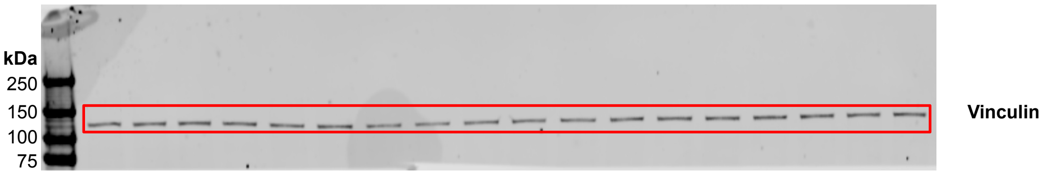

Extended Data Fig. 4a - anti-OXPHOS cocktail

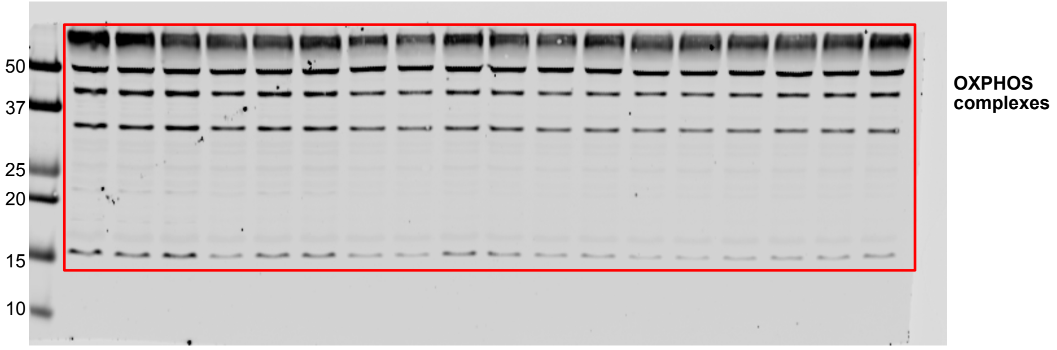

Extended Data Fig. 4d

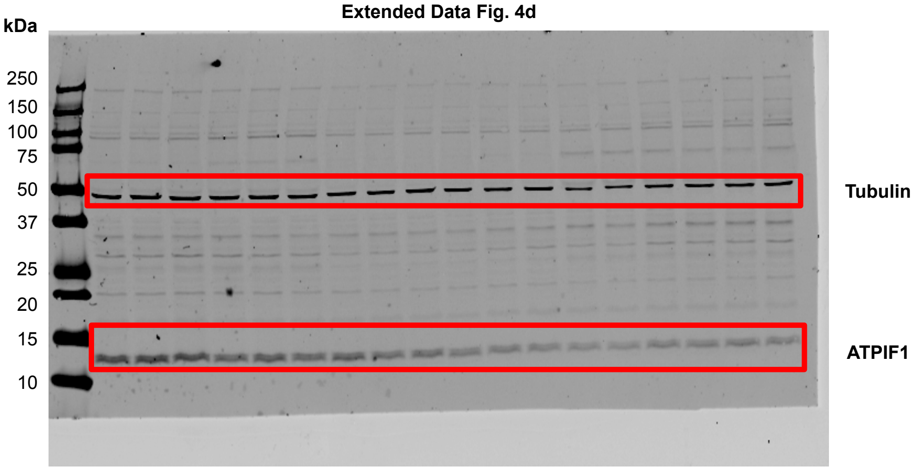

Extended Data Fig. 4d - anti-ATPIF1

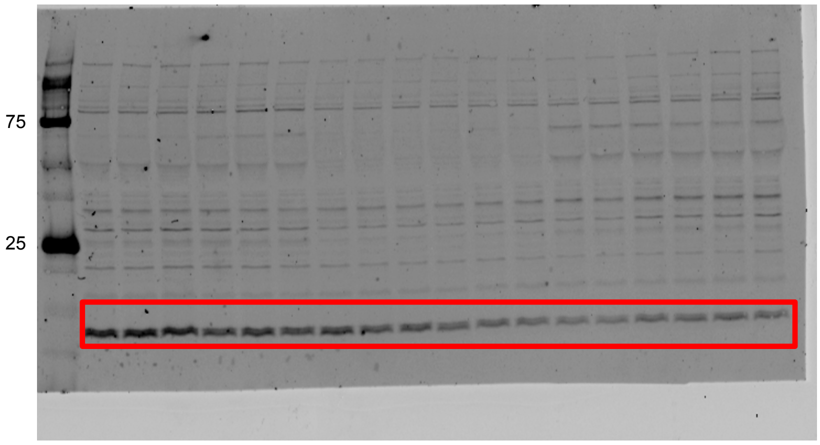

Extended Data Fig. 4d - anti-tubulin

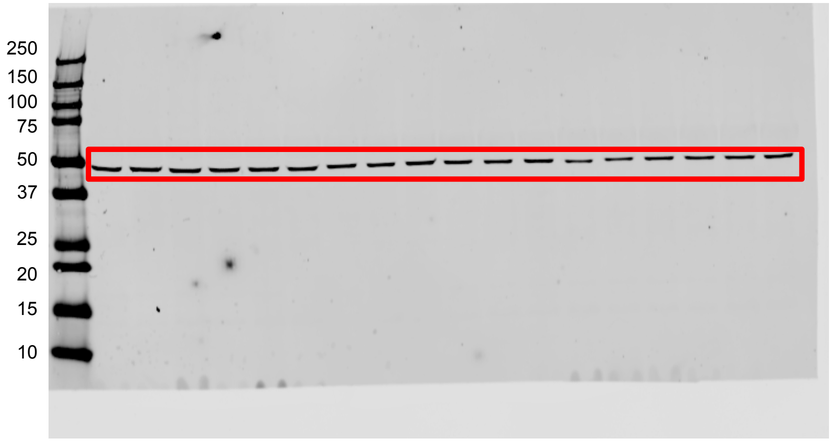

Supplement: Supplementary file 16 — Unprocessed western blots. [file 42255_2025_1224_MOESM16_ESM.pdf]
