# Supplementary material for: Pro-inflammatory macrophages produce mitochondria-derived superoxide by reverse electron transport at complex I that regulates IL-1β release during NLRP3 inflammasome activation
Source: Nat Metab. 2025 Feb 19;7(3):493–507. doi: 10.1038/s42255-025-01224-x (PMC11946910; doi:10.1038/s42255-025-01224-x)

Extended Data Fig. 10a

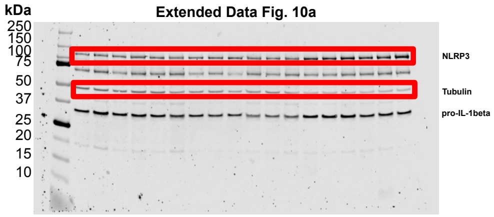

Extended Data Fig. 10a - anti-NLRP3 following anti-pro-IL-1beta

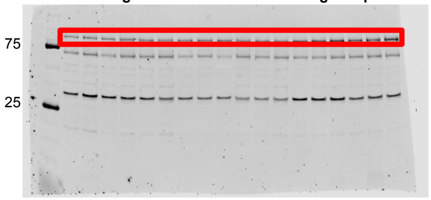

Extended Data Fig. 10a - anti-tubulin

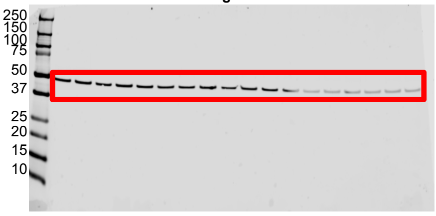

Extended Data Fig. 10c anti-pro-IL-1beta protein from supernatant (SN)

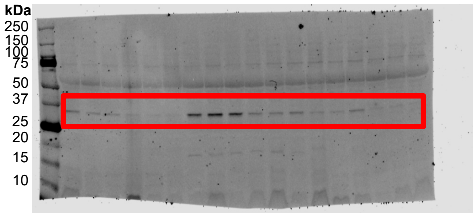

Extended Data Fig. 10c anti-pro-IL-1beta cell lysates

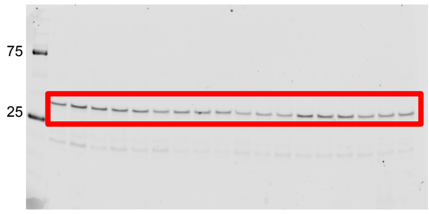

Extended Data Fig. 10c anti-cleaved IL-1beta protein from supernatant (SN)

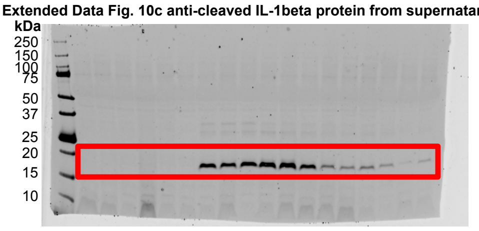

Extended Data Fig. 10c anti- tubulin cell lysates

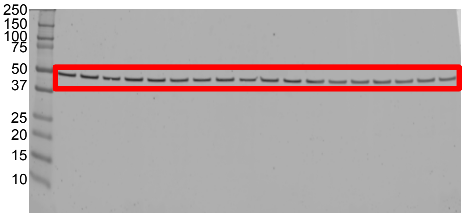

Extended Data Fig. 10g anti- caspase-1 p20 from SN

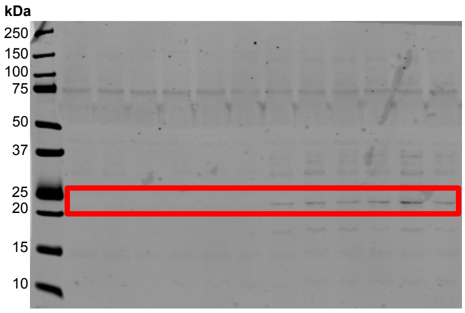

Extended Data Fig. 10g anti- caspase-1 p45 from cell lysate

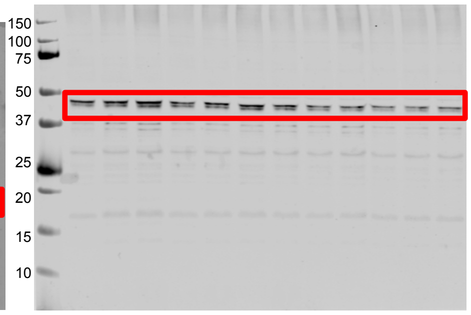

Extended Data Fig. 10g anti- vinculin from cell lysate

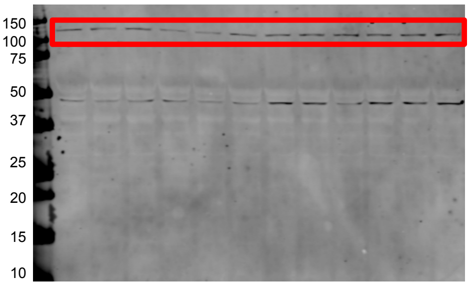

Extended Data Fig. 10j anti- ASC (Crosslinked)

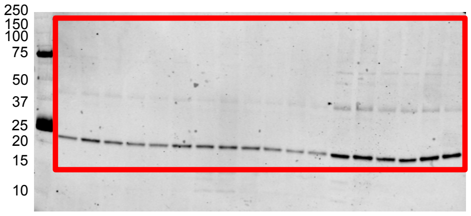

Extended Data Fig. 10j anti- ASC (input)

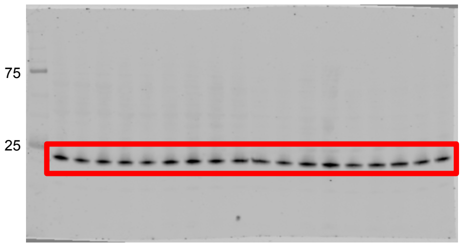

Extended Data Fig. 10j anti- tubulin (input)

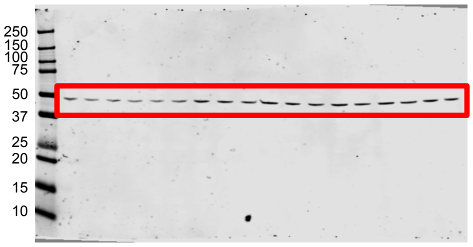

Extended Data Fig. 10k anti- GSDMD

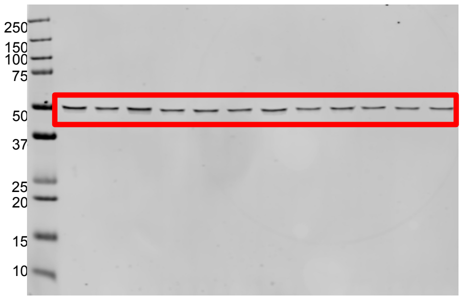

Extended Data Fig. 10k anti- Vinculin

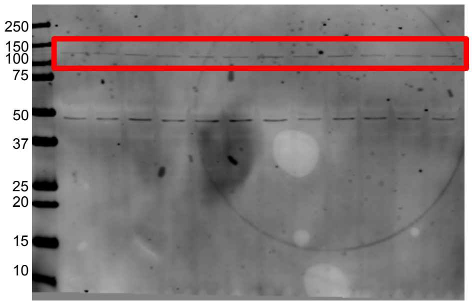

Extended Data Fig. 10k anti- cleaved GSDMD

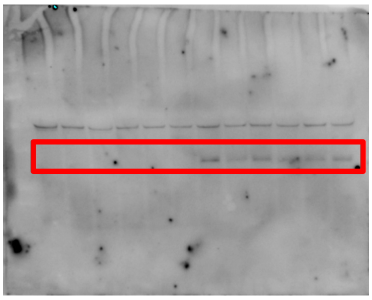

Extended Data Fig. 10k anti- cleaved GSDMD with marker

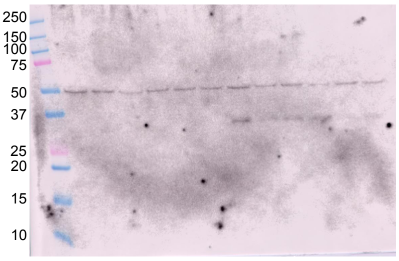

Supplement: Supplementary file 24 — Unprocessed western blots. [file 42255_2025_1224_MOESM24_ESM.pdf]
